# Pathways of China's PM_2.5_ air quality 2015–2060 in the context of carbon neutrality

**DOI:** 10.1093/nsr/nwab078

**Published:** 2021-04-29

**Authors:** Jing Cheng, Dan Tong, Qiang Zhang, Yang Liu, Yu Lei, Gang Yan, Liu Yan, Sha Yu, Ryna Yiyun Cui, Leon Clarke, Guannan Geng, Bo Zheng, Xiaoye Zhang, Steven J Davis, Kebin He

**Affiliations:** Ministry of Education Key Laboratory for Earth System Modelling, Department of Earth System Science, Tsinghua University, Beijing 100084, China; Ministry of Education Key Laboratory for Earth System Modelling, Department of Earth System Science, Tsinghua University, Beijing 100084, China; Department of Earth System Science, University of California, Irvine, CA 92697, USA; Ministry of Education Key Laboratory for Earth System Modelling, Department of Earth System Science, Tsinghua University, Beijing 100084, China; Ministry of Education Key Laboratory for Earth System Modelling, Department of Earth System Science, Tsinghua University, Beijing 100084, China; Chinese Academy of Environmental Planning, Beijing 100012, China; Chinese Academy of Environmental Planning, Beijing 100012, China; Ministry of Education Key Laboratory for Earth System Modelling, Department of Earth System Science, Tsinghua University, Beijing 100084, China; Joint Global Change Research Institute, Pacific Northwest National Laboratory, University Research Court, College Park, MD 20742, USA; Center for Global Sustainability, School of Public Policy, University of Maryland, College Park, MD 20742, USA; Center for Global Sustainability, School of Public Policy, University of Maryland, College Park, MD 20742, USA; State Key Joint Laboratory of Environment Simulation and Pollution Control, School of Environment, Tsinghua University, Beijing 100084, China; Institute of Environment and Ecology, Tsinghua Shenzhen International Graduate School, Tsinghua University, Shenzhen 518055, China; State Key Laboratory of Severe Weather & Key Laboratory of Atmospheric Chemistry of CMA, Chinese Academy of Meteorological Sciences, Beijing 100081, China; Department of Earth System Science, University of California, Irvine, CA 92697, USA; State Key Joint Laboratory of Environment Simulation and Pollution Control, School of Environment, Tsinghua University, Beijing 100084, China

**Keywords:** clean air policy, carbon neutrality, synergy pathway, air quality standards, WHO guidelines

## Abstract

Clean air policies in China have substantially reduced particulate matter (PM_2.5_) air pollution in recent years, primarily by curbing end-of-pipe emissions. However, reaching the level of the World Health Organization (WHO) guidelines may instead depend upon the air quality co-benefits of ambitious climate action. Here, we assess pathways of Chinese PM_2.5_ air quality from 2015 to 2060 under a combination of scenarios that link global and Chinese climate mitigation pathways (i.e. global 2°C- and 1.5°C-pathways, National Determined Contributions (NDC) pledges and carbon neutrality goals) to local clean air policies. We find that China can achieve both its near-term climate goals (peak emissions) and PM_2.5_ air quality annual standard (35 μg/m^3^) by 2030 by fulfilling its NDC pledges and continuing air pollution control policies. However, the benefits of end-of-pipe control reductions are mostly exhausted by 2030, and reducing PM_2.5_ exposure of the majority of the Chinese population to below 10 μg/m^3^ by 2060 will likely require more ambitious climate mitigation efforts such as China's carbon neutrality goals and global 1.5°C-pathways. Our results thus highlight that China's carbon neutrality goals will play a critical role in reducing air pollution exposure to the level of the WHO guidelines and protecting public health.

## Teaser

This study suggests that reducing PM2.5 exposure of the majority of the Chinese population to the WHO guideline (10ug/m3 for annual mean) will require ambitious low-carbon transition in energy system, highlighting the critical role of carbon neutrality goals in long-term air quality improvement in China.

## INTRODUCTION

Millions of people die every year from diseases caused by exposure to outdoor fine particulate matter (PM_2.5_) pollution [1,2], and more than a quarter of these premature deaths occur in China due to the country's severe air pollution [3–5]. In response to the country's air quality standard [6] for ambient PM_2.5_ (35 μg/m^3^ for annual mean concentration), substantial improvements in air quality have been observed since 2013, when a series of intensive air pollution control policies were implemented [7,8]. Yet, ∼80% of the Chinese population are still exposed to annual mean concentrations of PM_2.5_ that exceed 35 μg/m^3^, and >99% of the population are exposed to concentrations in excess of the World Health Organization (WHO) Air Quality Guidelines of 10 μg/m^3^ [3,4,9,10]. Moreover, China's aging population will be increasingly sensitive to PM_2.5_ air pollution in the future, so that the health impacts of that pollution are expected to rise over time [11]. Protecting public health by meeting the standards of the WHO Air Quality Guidelines thus calls for even more ambitious clean air actions [10,12].

Besides air pollution control policies, climate actions aiming to reduce fossil fuel consumption also have substantial air quality benefits [13]. In September 2020, China announced its ambitious climate commitment to achieve carbon neutrality by 2060, which may be the means by which long-term air quality improvement is brought about in China. Previous studies have shown that clean air and climate policies can each have large benefits on China's air quality in the future [14–21]. However, none of these studies have incorporated China's clean air action since 2013 together with China's new climate goal (i.e. carbon neutrality) and/or current international climate targets (i.e. 1.5°C and 2°C warming limits) to investigate the role of those ambitious climate policies in long-term air quality improvement. For one thing, many studies [14–19] use air pollution scenarios from global databases (e.g. the GAINS database and ScenarioMIP for CMIP6) that do not reflect the clean air policies and air quality improvements that have occurred in China since 2010 [7,8]. For another, the few forward-looking studies [20,21] that include recent changes in local air pollution are disconnected from long-term climate pathways. Specifically, the investigation of China's long-term PM_2.5_ air quality pathway in the context of carbon neutrality is missing. Hence, it is not clear how local clean air policies and China's two-stage climate mitigation efforts (i.e. National Determined Contributions (NDC) pledges and carbon neutrality goals) may interact in the future, which is preventing a realistic roadmap of China's long-term air quality.

Here, we combine a China-focused Integrated Assessment Model (IAM; GCAM-China) [22,23], a technology-based emission projection model (Dynamic Projection for Emission in China, DPEC) [24] and a chemical transport model (Weather Research and Forecasting, and Community Multiscale Air Quality, WRF-CMAQ) [25,26] to dynamically evaluate the potential for air quality improvements in China under a range of different climate and clean air policy pathways (Fig. S1). Specifically, we use the GCAM-China model to project the evolution of China's energy system (Fig. S2) under China's carbon neutrality goals and the global climate-socioeconomic scenarios from the Coupled Model Intercomparison Project Phase 6 (CMIP6) [27,28]. Interacting with local policy-based pollution control measures (Table S1), a group of emission scenarios are designed linking different climate goals with national clean air actions (Table 1 and Table S2). Therein, the *Baseline* scenario is designed with an unambitious climate target (i.e. RCP6.0) and no new clean air policies on the basis of 2015 levels. A policy-based *Current-goals* scenario would meet medium-term (i.e. 2030) air quality targets by meeting the NDC pledges and implementing released clean air actions (Table S1). Focusing on longer-term (i.e. 2060, the target year of carbon neutrality) air quality improvement, four additional policy scenarios reflect ambitious pollution control measures (Table S1) under different national and global climate goals (i.e. *Ambitious-pollution-NDC-goals*, *Ambitious-pollution-Neutral-goals*, *Ambitious-pollution-2°C-goals*, and *Ambitious-pollution-1.5°C-goals*). We then use the DPEC model to dynamically project China's future anthropogenic emission pathways under the scenario ensembles (Fig. S3). Finally, we utilize WRF-CMAQ chemical transport simulations and a hindcast of PM_2.5_ historical datasets [10] to estimate future total PM_2.5_ exposure (i.e. anthropogenic and natural sources) in China under a wide range of energy and anthropogenic emission trajectories. Further details of these scenarios and the simulation of pollution concentrations are provided in Methods and Supplementary Data.

**Table 1.** Summary of scenarios used in this study. A detailed description of different scenarios can be found in Supplementary Data (Tables S1 and S2).

Scenario	Definition	Climate constraints	Socioeconomic drivers	End-of-pipe pollution control
*Baseline*	*Baseline* offers the reference point for other scenarios. Following the SSP4 highly inequal and isolated mode, the climate constraints are negligible under RCP6.0 and the environmental control would remain at the 2015 level.	RCP6.0	SSP4	Same as 2015 level.
*Current-goals*	*Current-goals* presumes China will achieve its NDC pledges and the national PM_2.5_ air quality standard (i.e. 35 μg/m^3^) by 2030, elucidating China's future air pollution mitigation pathway towards all the released and upcoming clean air policies since 2015.	RCP4.5	SSP2	Current released and upcoming policies.
*Ambitious-pollution-NDC-goals*	*Ambitious-pollution-NDC-goals* shares the same energy and socioeconomic developments with the *Current-goals* scenario, but would fully deploy the best available end-of-pipe control technologies across all sectors by 2050, and implement them consistently between 2050 and 2060.	RCP4.5	SSP2	Best available end-of-pipe pollution control technologies.
*Ambitious-pollution- Neutral-goals*	*Ambitious-pollution-Neutral-goals* is designed to pursue China's carbon neutral commitment and the WHO PM_2.5_ guideline (i.e. 10 μg/m^3^) in long-term air quality improvement by 2060. It shares the same end-of-pipe control with the *Ambitious-pollution-NDC-goals* scenario, but more ambitious climate policies would be implemented to ensure China's net-zero CO_2_ emissions in 2060.	China's net-zero CO_2_ emissions in 2060	SSP1	Best available end-of-pipe pollution control technologies.
*Ambitious-pollution-2°C-goals*	*Ambitious-pollution-2°C-goals* is designed to pursue the 2°C temperature limits and the WHO PM_2.5_ guideline (i.e. 10 μg/m^3^) in long-term air quality improvement by 2060. It shares the same end-of-pipe control with the *Ambitious-pollution-NDC-goals* scenario, but more ambitious 2°C-consistent climate policies would be implemented.	RCP2.6	SSP1	Best available end-of-pipe pollution control technologies.
*Ambitious-pollution-1.5°C-goals*	*Ambitious-pollution-1.5°C-goals* is designed to pursue the 1.5°C temperature limits and the WHO PM_2.5_ guideline (i.e. 10 μg/m^3^) in long-term air quality improvement by 2060. It shares the same end-of-pipe control with the *Ambitious-pollution-NDC-goals* scenario, but more ambitious 1.5°C-consistent climate policies would be implemented.	RCP1.9	SSP1	Best available end-of-pipe pollution control technologies.

## RESULTS

**Figure 1.**
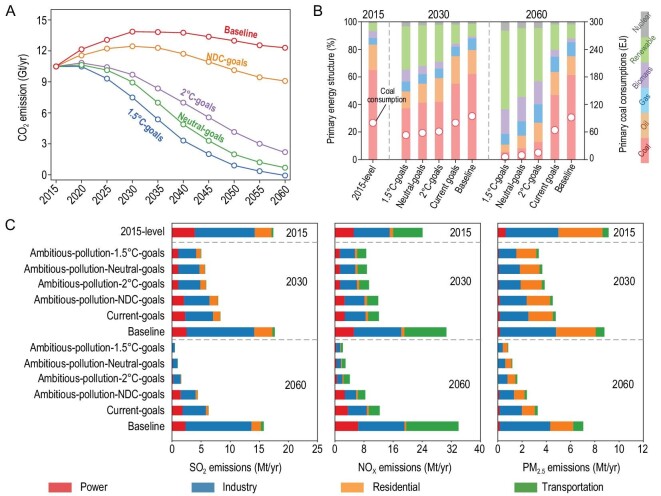
Future anthropogenic emission pathways and energy transitions over China. (A) Anthropogenic CO_2_ emissions between 2015 and 2060. (B) Primary energy structure (stacking histogram with the left Y-axis) and coal consumption (red circles with the right Y-axis) in 2015, 2030 and 2060. (C) Future air pollutant emissions (SO_2_, NO_x_ and primary PM_2.5_) by sector in 2015, 2030 and 2060 under different mitigation pathways.

Given current clean air policies and NDC pledges (i.e. *Current-goals*), the share of China's primary energy derived from coal steadily decreases from 65% in 2015 to 48% in 2060 (Fig. 1B). Consequently, annual anthropogenic CO_2_ emissions peak in 2030 at 12.4 Gt (18% higher than the 2015 level), and then fall back to 9.1 Gt in 2060 (13% lower than the 2015 level; orange curve in Fig. 1A), driven by low-carbon energy transitions in power and industry sectors (Fig. S4). Meanwhile, 46%–52% of China's pollutant emissions would be eliminated by 2030 under these current policies compared to the 2015 level, driven by changes in the industry and power sector transitions for all pollutants, and in the transportation sector for NO_x_ emissions (Fig. 1C, Fig. S3). However, further reductions after 2030 are modest without new clean air and climate actions: CO_2_ decreases by 27% and other pollutant emissions fall by only 4%–31% between 2030 and 2060.

In contrast, the combination of carbon neutral climate policies and very strict pollution control polices (i.e. *Ambitious-pollution-Neutral-goals*) leads to 20% lower fossil fuel fraction relative to the NDC goals scenario (i.e. *Current-goals* and *Ambitious-pollution-NDC-goals*; Fig. 1B) over the period 2015–2030, resulting in a reduction of 15% in CO_2_ emissions (green curve in Fig. 1A) and 58%–67% in pollution emissions between 2015 and 2030. Again, these reductions are mainly in the power and industry sectors, as well as in the transportation sector for NO_x_ emissions (Fig. 1C). But more importantly, large emission reductions continue after 2030 under the pathway to carbon neutrality goals. Annual anthropogenic CO_2_ emissions decrease to 0.68 Gt in 2060, reaching net-zero CO_2_ emissions accompanied by ∼0.7 Gt CO_2_ natural carbon sink (see Table S2 and S3). Between 2030 and 2060, along with 92% of CO_2_ reductions driven by the large decrease in total coal consumption and its fractions in primary energy (i.e. decreasing by 84% and 33%, respectively; Fig. 1B), 67%–83% pollution emission reductions can be further achieved. In addition to the main contribution to emission reductions from the power and industry sectors, transportation and residential sectors also significantly lower NO_x_ and PM_2.5_ emission.

When compared with global climate ambitions, we find that the CO_2_ emission pathway under China's carbon neutrality goals (i.e. *Ambitious-pollution-Neutral-goals*) lies between the 1.5°C- and 2°C-consistent climate policies (i.e. *Ambitious-pollution-2°C-goals* and *Ambitious-pollution-1.5°C-goals*). Compared with carbon neutrality goals, a more aggressive global climate target of 1.5°C leads to a 10% lower fossil fuel fraction in national primary energy structure in 2060—further reduces 0.45 Gt CO_2_ emissions (green and blue curves in Fig. 1A; Fig. S5) and 20%–50% of pollution emissions (Fig. 1C) in 2060. In contrast, 2°C-consistent climate policies lead to 1.2 Gt higher CO_2_ emissions and 29%–61% more pollution emissions in 2060 compared to carbon neutrality goals (Fig. S5; Fig. 1C). It is noticed that CO_2_ emissions are negative in 2060 under the 1.5°C climate ambition (−0.07 Gt, *Ambitious-pollution-1.5°C-goals*), in contrast to considerable air pollution emissions. This is because wide application of carbon capture and storage technologies, as well as negative carbon technologies, could absorb ∼1.26 Gt CO_2_ in 2060, thus neutralizing all CO_2_ emissions from fossil fuel facilities (∼1.19 Gt; Fig. S5). However, those facilities still release air pollutant emissions.

**Figure 2.**
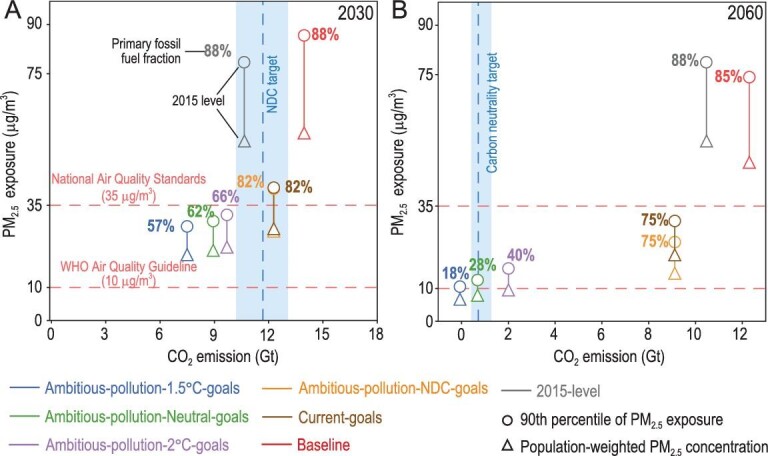
Accessibility of future climate targets and air quality improvements over China. Estimates of future CO_2_ emissions and PM_2.5_ exposure under different mitigation pathways in (A) 2030 and (B) 2060. The circle and triangle markers represent the 90th percentile of PM_2.5_ exposure and the population-weighted PM_2.5_ concentration, respectively. Labeled percentage numbers refer to the fossil fuel fraction in the primary energy mix. The horizontal red dashed lines represent the National Ambient Air Quality Standards (i.e. 35 μg/m^3^) and WHO Air Quality Guideline (i.e. 10 μg/m^3^). Light blue shaded portions are the ranges of published and simulated results on China's anthropogenic CO_2_ emission peak under the NDC target (in panel A) and the projected natural carbon sink in 2060 under low radiation forcing scenarios (in panel B). The dark blue dashed line represents the mean value of collected data.

Both climate policies and clean air actions that aim to eliminate pollution emissions can consequently improve air quality. Figure 2 shows PM_2.5_ exposure and carbon emissions in 2030 (Fig. 2A) and 2060 (Fig. 2B). Specifically, we select both national population-weighted mean PM_2.5_ concentration and the 90th percentile of PM_2.5_ exposure to access the future PM_2.5_ exposure. Meanwhile, CO_2_ emission pathways compatible with China's commitments to the NDC pledges and carbon neutrality goals are also shown as the carbon target indicators (blue shaded area; Table S3). We use a range of estimates on China's natural carbon sink in 2060 [29], under the low radiation forcing scenarios (i.e. RCP2.6, RCP1.9), as the carbon neutrality indicators.

**Figure 3.**
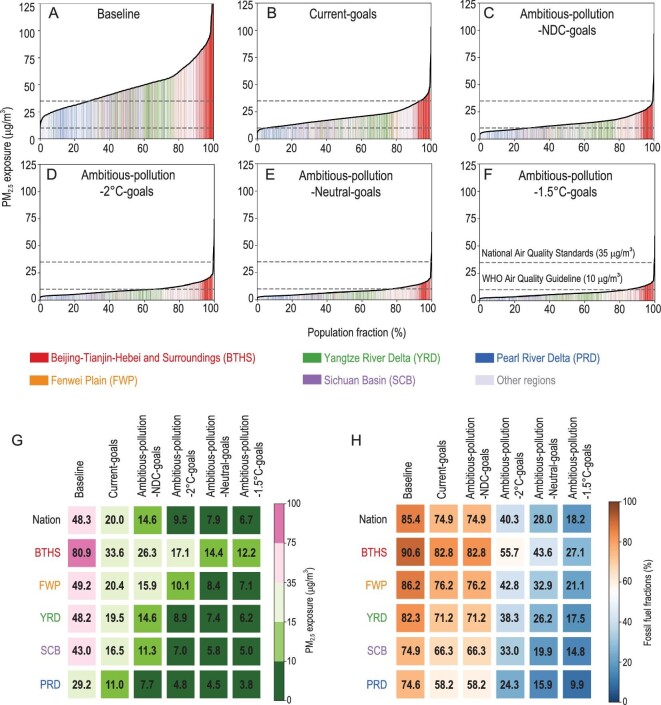
Regional disparities of future PM_2.5_ exposure and energy evolutions. (A–F) Accumulated PM_2.5_ exposure by 0.1° × 0.1° grid in 2060 under different scenarios (ranked from low to high, colored by region; results for 2030 are shown in Fig. S6). The horizontal gray dashed lines represent the national ambient air quality standards (i.e. 35 μg/m^3^) and WHO Air Quality Guideline (i.e. 10 μg/m^3^). (G) National and regional population-weighted mean PM_2.5_ concentration in 2060. (H) Fossil fuel fraction in primary energy mix in 2060.

With lax climate and clean air policies (i.e. *Baseline*), China will not meet either its current climate goals or air quality standards. Rather, in 2030 CO_2_ emissions will be 16% higher than its NDC pledge (blue dotted line in Fig. 2A) and population-weighted PM_2.5_ exposure will be 63% greater than its current standard of 35 μg/m^3^ (Fig. S7). With the combination of current pledges and policies (i.e. *Current-goals*), China's CO_2_ emissions will peak and its current PM_2.5_ goals will be achieved in 2030 (i.e. population-weighted PM_2.5_ exposure decreases by 50% to 27.6 μg/m^3^, with 79% of the population below the standard of 35 μg/m^3^). However, these current policies (i.e. *Current-goals*) deliver only small air quality improvements after 2030: the population-weighted PM_2.5_ exposure in 2060 is 20.2 μg/m^3^—more than double the WHO guideline of 10 μg/m^3^ (with almost 94% of the population, 1.2 billion people, exposed to pollution concentrations above the WHO guideline; Fig. 3B). Even under stricter clean air policies (i.e. *Ambitious-pollution-NDC-goals*), by 2060, 72% of the total population (918.2 million people) are still exposed to PM_2.5_ pollution that exceeds the WHO guideline (Fig. 3C).

Of the scenarios we analyze, only those that couple strict clean air policies with ambitious climate policies reach the low pollution levels endorsed by the WHO by 2060. With the strict clean air policies and more aggressive climate targets (*Ambitious-pollution-2°C-goals, Ambitious-pollution-Neutral-goals* and *Ambitious-pollution-1.5°C-goals*), additionally phasing out 16%–25% of fossil fuel fractions by 2030 (compared to *Current-goals*)—cutting CO_2_ emissions by 2.7–4.9 Gt—improves the air quality to well below 25 μg/m^3^ in 2030 (20.0–22.3 μg/m^3^). Moreover, air quality could continue to be improved after 2030. By 2060, non-fossil-fuel-dominated energy structures (18%–40% of primary fossil fuel) together with strict clean air policies would drive PM_2.5_ concentration well below the WHO guideline of 10 μg/m^3^ (9.5, 7.9 and 6.7 μg/m^3^, respectively, in *Ambitious-pollution-2°C-goals, Ambitious-pollution-Neutral-goals* and *Ambitious-pollution-1.5°C-goals*). Correspondingly, strict clean air policies plus carbon neutrality goals (*Ambitious-pollution-Neutral-goals*) could mean that 78% of the population (943.6 million people) are exposed to below 10 μg/m^3^. The best co-control pathway that accords with the global 1.5°C-consistent scenario (*Ambitious-pollution-1.5°C-goals*) could ensure that an extra 7% of the population (84.9 million people) are exposed to below 10 μg/m^3^ compared to the carbon neutrality pathway. Thus, ambitious low-carbon transitions in strict climate targets play critical roles in long-term air pollution exposure improvement. Regional differences in the spatial pattern of China's PM_2.5_ pollution (Fig. S6) and corresponding differences in energy structure reveal diverse challenges in energy transitions and realizable air quality improvements. We select five heavily polluted and densely populated key regions (i.e. Beijing-Tianjin-Hebei and Surroundings (BTHS), Fenwei Plain (FWP), Yangtze River Delta (YRD), Sichuan Basin (SCB) and Pearl River Delta (PRD); for region definitions see Table S4) to explore the regional patterns in PM_2.5_ exposure under different pathways. Figure 3 shows the regional disparities of future PM_2.5_ exposure and evolution in energy structure by 2060 (results in 2030 are shown in Fig. S7). As the most heavily polluted region, BTHS gathers almost the top 10% highest PM_2.5_-exposed population no matter which clean air and climate mitigation policies are implemented (Fig. 3A–F). Specifically, the carbon neutrality pathways plus strict clean air policies could only drive the PM_2.5_ air pollution exposure in the BTHS region to 14.4 μg/m^3^ by 2060 (*Ambitious-pollution-Neutral-goals*), which is still 44% higher than the WHO guideline. More strikingly, more than 60% of the population of China who are exposed to levels of pollution above the WHO guideline live in the BTHS region in 2060, under the *Ambitious-pollution-Neutral-goals* scenario.

Heavy air pollution in the BTHS region is mainly driven by the large emissions from heavy industry and the residential sector (both now fueled by fossil fuels) [30–32], together with unfavorable topography and meteorological conditions [33,34]. Currently, Hebei province within the BTHS region accounts for 13% of global raw steel production [35]. The BTHS region also consumes 12% of the fossil fuels used by the residential sector in China, though only 8% of the country's population reside there. Together, these sectors pose more substantial transition challenges than those faced by other regions (Fig. 3H). Meanwhile, a slow transition to non-fossil energy sources in the BTHS region (e.g. only ∼46% fossil fuel reduction between 2015 and 2060 under *Ambitious-pollution-Neutral-goals*; Fig. 3H) poses enormous health burdens. Those difficult-to-decarbonize industries (e.g. iron and steel, and the cement industry) in the BTHS region would bring long-term air pollution and health threats. Substantial improvements in PM_2.5_ exposure for a large number of Chinese people who live in the polluted area thus rely heavily on accelerating the transition to low-carbon energy in the BTHS region.

## DISCUSSION

For the first time, our study investigates China's future PM_2.5_ air quality improvement pathway in the context of carbon neutrality. We demonstrate the critical importance of China's carbon neutrality goals if China is to achieve large reductions in air pollution exposure after 2030. In our scenarios, the benefits of end-of-pipe pollution control measures are mostly exhausted by 2030, and only the systemic changes in energy sources that accompany ambitious climate mitigation (i.e. carbon-neutral-consistent, 1.5°C-consistent and 2°C-consistent energy transitions) can ensure China reduces PM_2.5_ exposure to below WHO standards (<10 μg/m^3^) by 2060. China's carbon neutrality goals could mean the majority of the Chinese population (∼78%) will experience a PM_2.5_ exposure below 10 μg/m^3^ (Fig. 3E), and that the PM_2.5_ concentration of nearly 85% of cities will meet the WHO guideline by 2060 (Fig. S8). The impact on air quality improvement is larger than the 2°C-consistent pathway (which predicts that 66% of the population and 72% of cities are exposed to below 10 μg/m^3^), but weaker than 1.5°C-consistent energy transitions (which predicts that 85% of the population and 93% of cities are exposed to below 10 μg/m^3^). In contrast, prior studies [14–19] concluded that China's population-weighted PM_2.5_ concentration, which implements NDC transformations and current legislation derived from a global database, basically fails to achieve the PM_2.5_ air quality standard in 2030. The highest projection of these studies (i.e. 52 μg/m^3^) is almost twice of our estimation (∼28 μg/m^3^). Similarly, no prior studies have shown that levels of PM_2.5_ in China could meet the WHO guideline of 10 μg/m^3^ by the middle of the century (or even the WHO’s interim target of 15 μg/m^3^) even with the strictest climate constraints and pollution control measures. This is because we thoroughly incorporate China's current and upcoming clean air efforts in our projections (Table S1). These efforts were largely overlooked by previous studies, which makes the WHO standards seem out of reach.

Our study is subjected to several uncertainties and limitations. First, the base-year PM_2.5_ exposure estimates (i.e. PM_2.5_ Hindcast Dataset, PHD; see Methods for detail) are subject to uncertainty due to the multiple inputs and limited monitor coverage [10]. Previous studies indicated that the uncertainty ranges in annual mean PM_2.5_ exposure are within 17% [10,36]. Second, the WRF-CMAQ model simulation introduces uncertainties due to incomplete knowledge of chemical and physical processes and uncertain emission inventories [37,38]. Validation against surface observations shows that our model reasonably reproduces the spatial and temporal patterns of PM_2.5_ concentration across China (see Fig. S9). Besides, WRF-CMAQ is used to derive relative changes of PM_2.5_ exposures by comparing different scenario runs in this work, where the model uncertainties might be partially eliminated. Third, our policy-based mitigation pathways are based on existing knowledge of China's environmental policies and best available technologies. Future technical innovation aimed at end-of-pipe emissions might also profoundly improve future air quality, which is not included in our analysis. Fourth, carbon neutrality goals could be fulfilled by different energy pathways that lead to different air pollutant emission levels. In this work, we only demonstrate the air quality improvement from one specific carbon neutrality pathway, and more comprehensive analysis with different pathways should be investigated in the future. Last but not least, changes in future meteorological conditions and natural emissions induced by climate change are not considered in this study. Our simulation shows that natural emissions contributed ∼3.0 μg/m^3^ to China's population-weighted PM_2.5_ concentrations in 2015. However, as significant air quality improvements will be achieved from the anthropogenic emission reductions under the ambitious co-control pathways, the role of natural sources in total PM_2.5_ exposure will be more important in the future. Several studies have found that natural emissions [39,40] and meteorological conditions in the context of climate change [34,41] would exacerbate PM_2.5_ exposure in the future, which in turn calls for more vigorous action on climate change mitigation and air pollution control to protect public health. More detailed discussion on these uncertainties and limitations are presented in Supplementary Note 3.

The national PM_2.5_ air quality standard of 35 μg/m^3^ was first proposed in 2012 in recognition of the need to reduce PM_2.5_ air pollution to protect public health in China. However, as the country's population age in the future, the vulnerability to air pollution will markedly increase [42], such that the current national standard will no longer be sufficient to protect public health. The WHO guideline of 10 μg/m^3^ will therefore almost inevitably become the new long-term goal [9,12]. That is, we should expect that more ambitious air quality goals will prevail after 2030 to protect public health. Such substantial mitigation in the carbon neutrality context calls for indispensable energy transitions of more than 68% fossil fuel decrement, and respective coal and liquid fractions of less than 10% by 2060 (i.e. *Ambitious-pollution-Neutral-goals*)—even with the best available end-of-pipe pollution control measures. From the current policy scenario (i.e. *Current-goals*) to the best co-control pathway (i.e. *Ambitious-pollution-1.5°C-goals*), emission reductions are mainly concentrated in the coal-fired industry, power, residential sectors, oil-fired industry and transportation sectors (Fig. S10), which again demonstrates the crucial role of low-carbon energy transitions in promoting substantial air quality improvements. The current institutional set-up creates enabling conditions for developing and implementing synergistic clean air policies and climate mitigation actions, as both policies are currently managed by the Ministry of Ecology and Environment of the People's Republic of China.

Thus, our findings suggest that China's efforts to improve its air quality will entail a thorough shift from end-of-pipe-oriented to climate-mitigation-oriented emission reductions. Such a transition will likely be necessary in other developing regions with severe air pollution, such as India. This is different from the pathway followed by most developed regions (e.g. the US and European Union), where air pollution problems have been essentially solved through the highly efficient end-of-pipe controls of fossil fuel-burning technologies [43,44]. This is because pollution from coal combustion in more developed regions was mostly from large point sources such as power-generation and industry, which were relatively concentrated and easily controlled [43–45]. In contrast, in the most heavily polluted regions in developing countries (e.g. the BTHS in China and the Gangetic Plain in India), air pollution is dominated by small, widely scattered facilities and residential coal-burning devices; and the end-of-pipe control of these devices is considerably hindered [31,46–48]. Our analysis thus points to climate mitigation and decarbonization of the Chinese energy system as being critical to the long-term improvement of Chinese air quality and public health.

## MATERIALS AND METHODS

Figure S1 shows the methodology framework for this study. Three sub-sections, namely emission projection, air quality modeling and PM_2.5_ exposure estimation, are combined to investigate China's future PM_2.5_ exposure pathways. Firstly, we apply the GCAM-China model to simulate China's future energy and socioeconomic evolution under China's national carbon neutrality goals and a family of climate-socioeconomic scenarios within CMIP6 [27,28]. We then develop three air pollution control scenarios with a full consideration of China's clean air actions. Driven by energy, socioeconomic evolution and environmental policies, we project China's future emission pathways with the DPEC model. We build the WRF-CMAQ system to simulate future ambient PM_2.5_ concentrations over China, and finally estimate the future PM_2.5_ exposure based on the observation-based PM_2.5_ dataset, CMAQ-simulated PM_2.5_ concentrations and future demographics.

### Scenario design

Socioeconomic development, climate policy and pollution control actions are three principal influences on future anthropogenic emission variations. Based on our previous work [24], we design and extend China's future emission scenarios from 2015 to 2060 according to the three abovementioned aspects. For energy and economic assumptions, we firstly select four scenarios (SSP1–19, SSP1–26, SSP2–45 and SSP4–60) from the CMIP6 scenario matrix [27], which combines different Shared Socioeconomic Pathways (i.e. SSPs) and climate output based on Representative Concentration Pathways (i.e. RCPs) basically covering the range of climate-forcing levels and representing most possible energy patterns of future China. Under the context of China's carbon neutrality commitment, we further design a carbon neutral scenario that supposes China will achieve net-zero CO_2_ emissions in 2060, and combine this with the SSP1 sustainable socioeconomic assumptions. Based on China's near-term (i.e. by 2030) clean air measures and long-term plans, we establish three pollution control scenarios, namely *baseline*, *current goals* and *ambitious pollution control*, to narrate different pollution control levels. The detailed clean air measures (including both end-of-pipe control and technology turnover) and policy parameters of these three pollution control scenarios are listed in Table S1. We finally combine and develop a total of six emission scenarios (*Ambitious-pollution-1.5°C-goal*, *Ambitious-pollution-2°C-goal*, *Ambitious-pollution-Neutral-goal*, *Ambitious-pollution-NDC-goal*, *Current-goals* and *Baseline*) to investigate China's potential future air quality pathways. Details of the scenario ensembles and their relationship with Tong *et al.* [24] are described in Supplementary Note 1 and Tables S1–2.

### China's future energy and emission projections

China's future energy pathways under the scenario ensembles (Fig. S2) are generated with the China-focused version of the GCAM (GCAM-China) [22,23]. The GCAM-China model is an IAM with subnational information of China. China's future anthropogenic emissions are projected by the DPEC model [24]. The DPEC is composed of the energy, socioeconomic projection module, the combustion/production technology projection module and the end-of-pipe control technology turnover module. The first module is coupled with GCAM-China simulations through source mappings and base-year harmonization, and the latter two are developed with a set of technology-based turnover models. More detailed information on the DPEC model is described in Tong *et al.* [24]. Pathways of major air pollutant emissions (including SO_2_, NO_x_, PM_2.5_, NMVOC and NH_3_) under the scenario ensembles are shown in Fig. S3.

### Air quality modeling with the WRF-CMAQ model

We use Weather Research and Forecasting Version 3.9.1 [25] and Community Multiscale Air Quality Version 5.2.1 [26] to establish the air quality modeling system for China’s mainland in this work. The WRF-CMAQ simulation domain covers the mainland of China with a resolution of 36 km × 36 km horizontally and 14 layers vertically. Detailed configurations for the WRF-CMAQ modeling system are described in Supplementary Note 2.

Our study contains two experimental parts, namely core scenario and sensitivity simulations. In core scenario simulations, meteorological conditions and natural emissions are fixed at the 2015 level; future anthropogenic emissions of China and other countries under the scenario ensembles are derived from the DPEC model and CMIP6 emission database, respectively. We design two sets of sensitivity simulations to quantify the potential impact of meteorology change and global pollution transport on China's future PM_2.5_ evolution (Table S5). We collect the hourly observed PM_2.5_ concentration data from 1664 national observation monitors (China Environmental Monitor Center), and evaluate our base-year annual mean PM_2.5_ simulation by grids (Fig. S9). The comparisons generally show good agreement between annual mean PM_2.5_ simulations and observations.

### Future PM_2.5_ exposure estimates

Future PM_2.5_ exposure is estimated with future ambient PM_2.5_ concentrations and demographics. In this study, future ambient PM_2.5_ concentration is estimated by the combination of an observation-based PHD and WRF-CMAQ simulated ambient PM_2.5_ variations. The PHD product is developed by Tsinghua University and provides historical PM_2.5_ estimates between 2000 and 2016 across China [10]. The PHD assembles multiple data sources (e.g. the satellite retrieved aerosols, chemical transport model outputs and various spatiotemporal variables) through a machine-learning method, and hindcasts China's daily PM_2.5_ concentrations from 2000 to 2016. We then downscale all the CMAQ simulations into the 0.1° × 0.1° grid with the offline ordinary Kriging method to match the PHD spatially, and calculate the gridded ambient PM_2.5_ of future years with the base-year PHD dataset and future-year CMAQ-simulated variation ratios. The introduction of the PHD dataset can largely reduce the uncertainties of base-year PM_2.5_ estimations, and thus, future PM_2.5_ exposure evolutions that are induced by emission variations can be estimated more accurately.

The exposed population of the base year is derived from the fourth version of the Gridded Population of the World dataset (GPWv4), with a horizontal resolution of 1/120 degree (approx. 1 km). The variations of the future exposed population under the SSP scenarios are collected from the Inter-Sectoral Impact Model Comparison Project [49] (ISI-MIP population), with a resolution of 0.5 degree and a continuous time series from 2015 to 2100. These two population datasets are also re-gridded into 0.1° × 0.1° to match other datasets spatially. Future PM_2.5_ exposure of one particular region is finally estimated as the population-weighted average ambient PM_2.5_ concentration.

## DATA AVAILABILITY

Emission data (China's future emission scenarios 2015–2060) generated from this study are available at http://www.meicmodel.org/dataset-dpec.html.

## Supplementary Material

nwab078_Supplemental_File
