# Supplementary material for: Pathways of China's PM2.5 air quality 2015–2060 in the context of carbon neutrality
Source: Natl Sci Rev. 2021 Apr 29;8(12):nwab078. doi: 10.1093/nsr/nwab078 (PMC8692930; doi:10.1093/nsr/nwab078)
Supplement: nwab078_Supplemental_File [file nwab078_supplemental_file.pdf]

Supplementary Information for

Pathways of China's $PM_{2.5}$ air quality 2015-2060 in the context of carbon neutrality

Jing Cheng[1,#], Dan Tong[1,2,#], Qiang Zhang[1,*], Yang Liu[1], Yu Lei[3], Gang Yan[3], Liu Yan[1], Sha Yu[4], Ryna Yiyun Cui[5], Leon Clarke[5], Guannan Geng[6], Bo Zheng[6], Xiaoye Zhang[7], Steven J. Davis[2], and Kebin He[6]

[1]Ministry of Education Key Laboratory for Earth System Modelling, Department of Earth System Science, Tsinghua University, Beijing 100084, People's Republic of China

[2]Department of Earth System Science, University of California, Irvine, Irvine, CA 92697, USA

[3]Chinese Academy of Environmental Planning, Beijing 100012, China

[4]Joint Global Change Research Institute, Pacific Northwest National Laboratory, University Research Court, College Park, MD 20742, USA

[5]Center for Global Sustainability, School of Public Policy, University of Maryland, College Park, MD 20742, USA

[6]State Key Joint Laboratory of Environment Simulation and Pollution Control, School of Environment, Tsinghua University, Beijing 100084, China

[7]Chinese Academy of Meteorological Sciences, China Meteorological Administration, Beijing 100081, China

[#]These authors contributed equally to this work.
[*]Correspondence to: Qiang Zhang
Email: qiangzhang@tsinghua.edu.cn

Supplementary Information Text

Supplementary Note 1. **Rationale and definition of scenario ensembles.** A subset of six scenarios (*Baseline*, *Current-goals*, *Ambitious-pollution-NDC-goals*, *Ambitious-pollution-2℃-goals*, *Ambitious-pollution-Neutral-goals*, and *Ambitious-pollution-1.5℃-goals*) are designed to dynamically project China's future emission and air quality pathways. The combinations of different SSP and RCP scenarios (i.e. SSP1-26, SSP2-45, SSP4-60) are selected according to the Scenario Model Intercomparison Project (ScenarioMIP) for CMIP6[1]. O'Neill et al describe ScenarioMIP's objectives, experimental design, and its relation to other activities within CMIP6 in detail[1]. In summary, O'Neill choose an SSP for each global average forcing pathway by taking into consideration of the possibility that the sensitivity of climate outcomes to SSP choice may be larger than anticipated. To account for that possibility, choices were based on one or, when compatible, more of the following goals: facilitate climate research; minimize differences in climate; and ensure consistency with scenarios that are most relevant to the IAM/IAV communities. Therefore, an experimental design has been identified consisting of eight alternative 21st century scenarios (i.e. SSP5-8.5, SSP3-7.0, SSP2-4.5, SSP1-2.6, SSP4-6.0, SSP4-3.4, SSP5-3.4-OS, SSPa-b) plus one large initial condition ensemble (SSP3-7.0) and a set of long-term extensions (SSP5-8.5-Ext, SSP5-3.4-OS-Ext, and SSP1-2.6-Ext), divided into two tiers defined by relative priority. Therefore, given the purpose of this study and China's current development mode, we select SSP4-6.0 as the baseline climate scenario, SSP2-45 as the NDC-consistent scenario, SSP1-26 as the global 2℃ limits-consistent scenario, SSP1-19 as the global global 1.5℃ limits-consistent scenario.

Baseline represents the reference of the study, which includes China's enacted clean air actions before 2015. Following an inequal global developing mode, economic growth is much slower and results in limited climate policies and clean air actions. Simulations of *Baseline* could elucidate the possible deterioration of future air qualities without any restriction of fossil fuels or pollution control.

Current-goals is designed with current released and upcoming climate target (i.e. the National Determined Contributions (NDC) target) and environmental policies (Table S1). For environment policies, this scenario incorporates all of China's released and upcoming air pollution control policies, and these policies would also be enhanced during 2020–2030 in terms of national air quality goals of building 'a beautiful China'. Besides, regional air pollution control diversities are also fully considered according to China's latest clean air actions, that three key regions, namely the Beijing-Tianjin-Hebei and surroundings (BTHS), the Fenwei Plain (FWP) and the Yangtze River Delta (YRD) are highlighted in this scenario. *Current-goals* represents a middle and policy-continued developing mode, whose simulation results could indicate future ambient $PM_{2.5}$ variations under all the current policies.

Ambitious-pollution-NDC-goals shares the same energy inputs with *Current-goals*, but would maximumly adopt highly-developed end-of-pipe control technologies by 2050, and implement consistently during 2050-2060. End-of-pipe pollution control policies in this scenario present the best available air pollution control process in China. From 2015 to 2030, the strictest clean air process in the BTHS region would be deployed all over China; while during 2030–2050, the best available end-of-pipe control and combustion/production technologies would be gradually penetrated in all sectors; and then, these best technologies would be continued to operate during 2050-2060 (Table S1). Simulation results of *Ambitious-pollution-NDC-goals* help to reveal whether China could achieve ultimate air quality improvements through current energy transitions along with the strictest pollution control measures.

Ambitious-pollution-Neutral-goals is designed under the context of carbon neutrality commitment released by China's government, that China aspires to achieve carbon neutrality by 2060. Rigorous low-carbon energy transitions would be rapidly deployed to ensure the zero carbon emissions in China by 2060. Meanwhile, it

combines the best available pollution control technologies (Table S1), and assumes the social economy would develop sustainably at a reasonably high pace (i.e. the SSP1 sustainable pathway).

Ambitious-pollution-2℃-goals and *Ambitious-pollution-1.5℃-goals* scenarios are further designed to better understand the role and the impacts of China's carbon neutrality goals, which are consistent with the global 2℃ and 1.5℃ temperature rising limits, respectively; and they share the same pollution control and socioeconomic development with *Ambitious-pollution-Neutral-goals*. Together with the *Ambitious-pollution-Neutral-goals* scenario, these three strict low-carbon scenarios manifest China's future $PM_{2.5}$ air quality under the strictest carbon and pollution co-control pathways. Besides, it is also informative for China's air quality co-benefit potentials under the national carbon-zero-adapted and the global 2℃-/1.5℃-consistent energy optimizations.

Supplementary Note 2. **Configurations of WRF-CMAQ modeling system.** In this work, we use the Weather Research and Forecasting Version 3.9.1 (WRFv3.9.1) and the Community Multiscale Air Quality Version 5.2.1 (CMAQv5.2.1) to establish the air quality modelling system for China mainland. WRF and CMAQ model provide the meteorological conditions and pollutant concentrations respectively. The horizontal domain covers the mainland of China with a resolution of 36 km × 36 km. The vertical resolution is designed as 23 sigma levels from surface to tropopause (about 100 mb) for WRF simulation (with 10 layers below 3-km), and then these 23 sigma levels are collapsed into 14 sigma levels (1.000, 0.995, 0.988, 0.980, 0.970, 0.956, 0.938, 0.893, 0.839, 0.777, 0.702, 0.582, 0.400, 0.200 and 0.000) for CMAQ model.

We choose the New Goddard shortwave radiation scheme[2], RRTM[3] longwave radiation scheme, the Kain-Fritsch cloud parameterization[4] (version 2), the ACM2 PBL scheme[5], the Pleim-Xiu land-surface scheme[6] and WSM6 cloud microphysics for WRF simulation; and adopt the Analysis nudging, observational nudging, soil nudging to improve the modelling fidelity. The surface roughness is corrected by increasing the friction velocity by 1.5 times to reduce the high biases in wind speed[7]. We apply the CB05 gas-phase and AERO6 particulate matter chemical mechanisms for CMAQ modelling. The meteorological initial and boundary conditions are derived from the final analysis data (FNL). The chemical initial and boundary conditions are interpolated from the dynamic outputs of GEOS-Chem model, which are driven by future gridded CMIP6 emissions. All the simulations are conducted throughout the whole year and with a one-month spin-up.

For emission inputs, we utilize the time factors (i.e. monthly, daily, hourly), spatial and species (including both particulate matter and volatile organic compounds) proxies of the MEIC model (Multiresolution Emission Inventory for China)[8] to transform DPEC-derived China's future anthropogenic emissions into CMAQ-required formats. Global emissions (except for China) are derived from the gridded global CMIP6 emission datasets[9]. For other natural source emissions, biogenic emissions are simulated with the Model of Emission of Gases and Aerosols from Nature (MEGAN v2.1)[10]; sea-salt and dust emissions are calculated online in the CMAQ model; open fire burning emissions are obtained from the fourth-generation global fire emissions database (GFED4)[11].

Our study contains two parts of experiments, namely core scenario and sensitivity simulations. In core scenario simulations, meteorological conditions and natural emissions are fixed as 2015 level; future anthropogenic emissions of China and other countries under the scenario ensembles are derived from DPEC model and CMIP6 emission database respectively. We design two sets of sensitivity simulations to quantify the potential impacts of meteorology change and global pollution transports on future China's $PM_{2.5}$ evolutions. Taking *Ambitious-pollution-2℃-goals* as the criterion, we fix China's anthropogenic emissions under *Ambitious-pollution-2℃-goals* in all sensitivity simulations. For meteorology change, we simulate China's future air quality with the meteorology from 2000 to 2018, and then quantify its impacts by comparing the results of emission-fixed, meteorology-varied simulations with the core *Ambitious-pollution-2℃-goals* (2015-meteorology) simulation. For pollution transports, we vary the chemical boundary and global future emissions with SSP1-19 (the lowest emissions) and SSP3-70 (the highest emissions) CMIP6

scenarios. We then quantify the transport impacts by comparing the results of China emission-fixed, global emission and chemical boundary-varied simulations with the core *Ambitious-pollution-2℃-goals* simulation. Emission, meteorology, and chemical boundary configurations of all simulations are summarized in Table S5. We collect the hourly observed $PM_{2.5}$ concentration data from 1664 national observation monitors (China Environmental Monitor Center), and evaluate our base-year annual mean $PM_{2.5}$ simulations by grids (Fig. S9). The comparisons generally show good agreements of annual mean $PM_{2.5}$ simulations and observations.

Supplementary Note 3. **Uncertainties and limitations.** The projected China's future $PM_{2.5}$ exposures in this study are subjected to several uncertainties in each model and step.

First, the base-year (i.e. 2015) $PM_{2.5}$ exposure estimates are uncertain due to the multiple inputs, limited temporal and spatial coverage of monitor sites[12,13]. These uncertainties have been evaluated in previous studies, that the relative prediction error of annual mean $PM_{2.5}$ exposure is within 17%, and the mean bias is 1.61 $\mu g/m^3$. The annually iterated cross-validation correlation coefficient with the in-situ observations is high as 0.749, also showing the good agreements[12,13]. This dataset might be the best-available $PM_{2.5}$ estimates over China with complete spatiotemporal coverage.

Secondly, the WRF-CMAQ modelling system would induce inherent uncertainties on ambient $PM_{2.5}$ concentration simulations, especially the simulated response of $PM_{2.5}$ concentrations to the emission reductions[14,15], which exists uncertainties on the future $PM_{2.5}$ exposure projections under different mitigation pathways. It is largely because of the incomplete understanding of the chemical formulations, products, rate coefficients, and reaction kinetics[16]. We evaluate our base-year simulations with the hourly observed $PM_{2.5}$ concentration data from China Environmental Monitor Center. The comparisons show good agreements of the $PM_{2.5}$ simulations and observations, that the average correlation coefficient of all monitor sites is high as 0.79 (Fig. S9), the average normalized mean bias (NMB) and normalized mean error (NME) are 8.5% and 31.4% respectively, well inside the recommended criteria for $PM_{2.5}$ photochemical model performance statistics (NMB<±30%, NME<50%, R>0.40)[17]. Besides, the pollution mitigation response to the emission reductions have been well simulated in our previous studies, and the simulated $PM_{2.5}$ concentrations and compositions can well capture the relevant observation changes[18,19].

Third, meteorology fluctuations[20] and global pollution transports[21] would influence the degree of China's future air quality improvements. Employing the meteorology of last two decades (Table S5), we find that the meteorology impacts are relatively small on China's future $PM_{2.5}$ exposure evaluations, with the fluctuation margin of -7% – 6% under *Ambitious-pollution-2℃-goals* pathway (Fig. S11). As for the global pollution transports, we find their impacts on China's future $PM_{2.5}$ exposure are also marginal, with the relative influences of -2%–6% and -1%–5% in 2030 and 2060 under *Ambitious-pollution-2℃-goals* pathway respectively.

Fourth, carbon neutrality goals could be fulfilled by different energy pathways which lead to different air pollutant emission levels. In this work, we only demonstrate the air quality benefit from one specific carbon neutrality pathway, and more comprehensive analysis with different pathways should be investigated in the future.

Our estimations are also subject to some limitations. On the one hand, the three air pollution scenarios are designed with existing knowledge of China's localized environmental policies and best-available technologies. Future technical innovation targeted end-pipe emissions are not included in our analysis. The unpredictable technical innovation on end-pipe pollution control, especially for non-energy related sources (i.e. agriculture, solvent use), might also profoundly benefit future air quality. On the other hand, future variations of meteorology[22-25] and natural source emissions[26-29] induced by climate change remain highly uncertain, but are not considered in this study, that they are fixed in the 2015 level in core simulations (Table S5). Several studies have demonstrated future meteorological conditions are likely to deteriorate air pollution through intense extreme events, enhanced thermal stability and the Arctic sea ice melting[22-25]. For example,

meteorology under the RCP4.5 climate pathway would increase China's population-weighted $PM_{2.5}$ concentrations by 3% in 2050[23]. In response to future climate and land cover change, previous studies concluded the natural dust loading would vary from -19%–9% (vs. modern natural emissions) globally[26]; and the biogenic VOC emissions in China would increase by 11.13% and 25.20% under RCP4.5 and RCP8.5 climate scenarios respectively in 2050[29]. Our simulation shows the natural source emissions contribute ~3.0 $\mu g/m^3$ to China's population-weighted $PM_{2.5}$ concentrations in 2015. However, as the significant air quality improvements will be achieved from the anthropogenic emission reductions under the ambitious co-control pathways, the role of natural sources in total $PM_{2.5}$ exposure will be more highlighted. And given these climate change-induced natural emissions and meteorological conditions might exacerbate $PM_{2.5}$ exposure in the future, more mitigation efforts are needed to better protect public health.

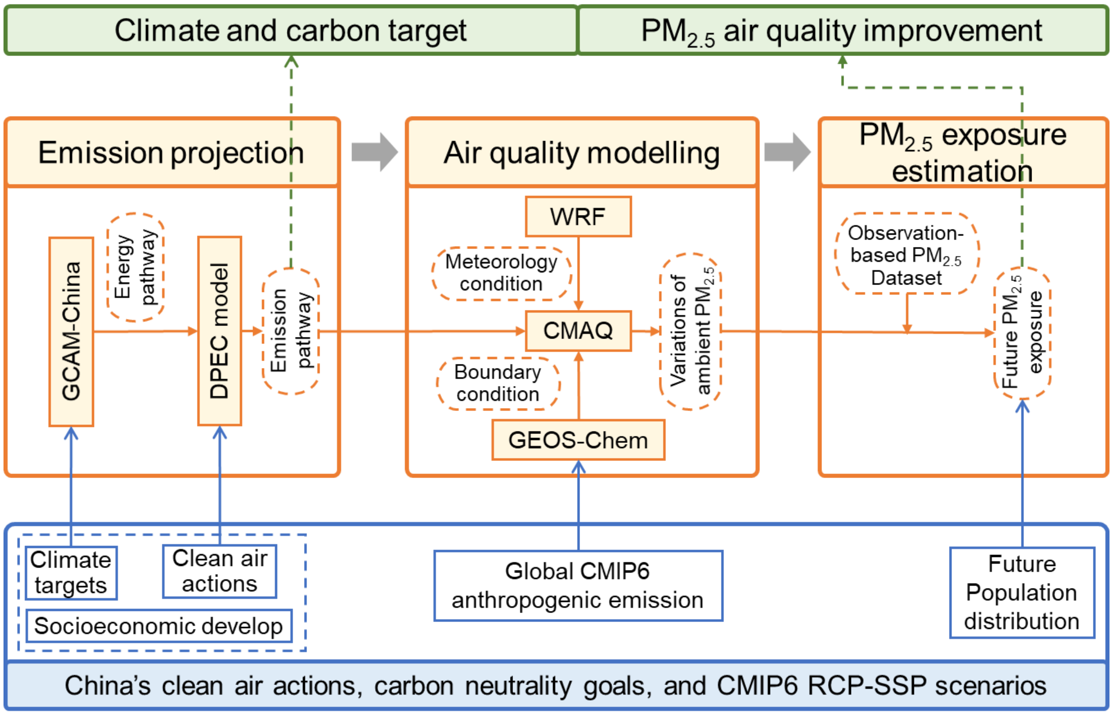

148 **Fig. S1.** The methodology framework.

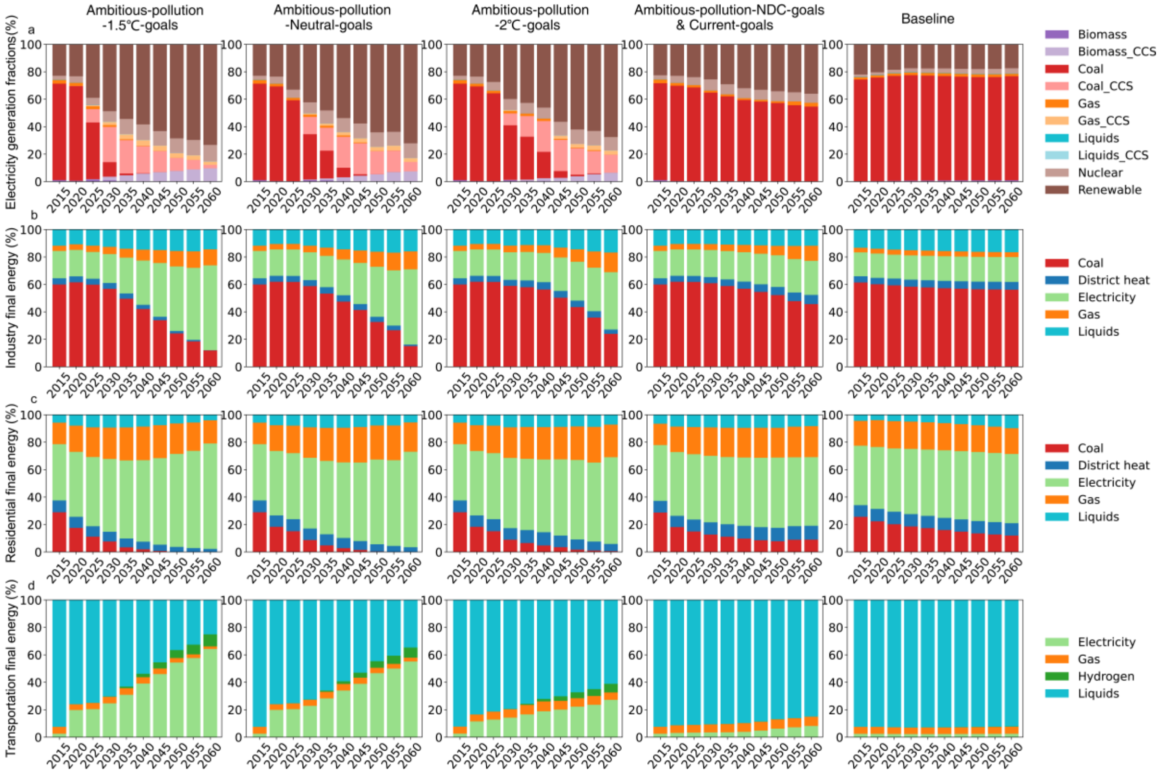

Fig. S2. Future energy evolutions under different scenarios during 2015-2060. (a) Electricity generation pathways. (B) Industry final energy transitions. (c) Residential final energy transitions. (d) Transportation final energy transitions.

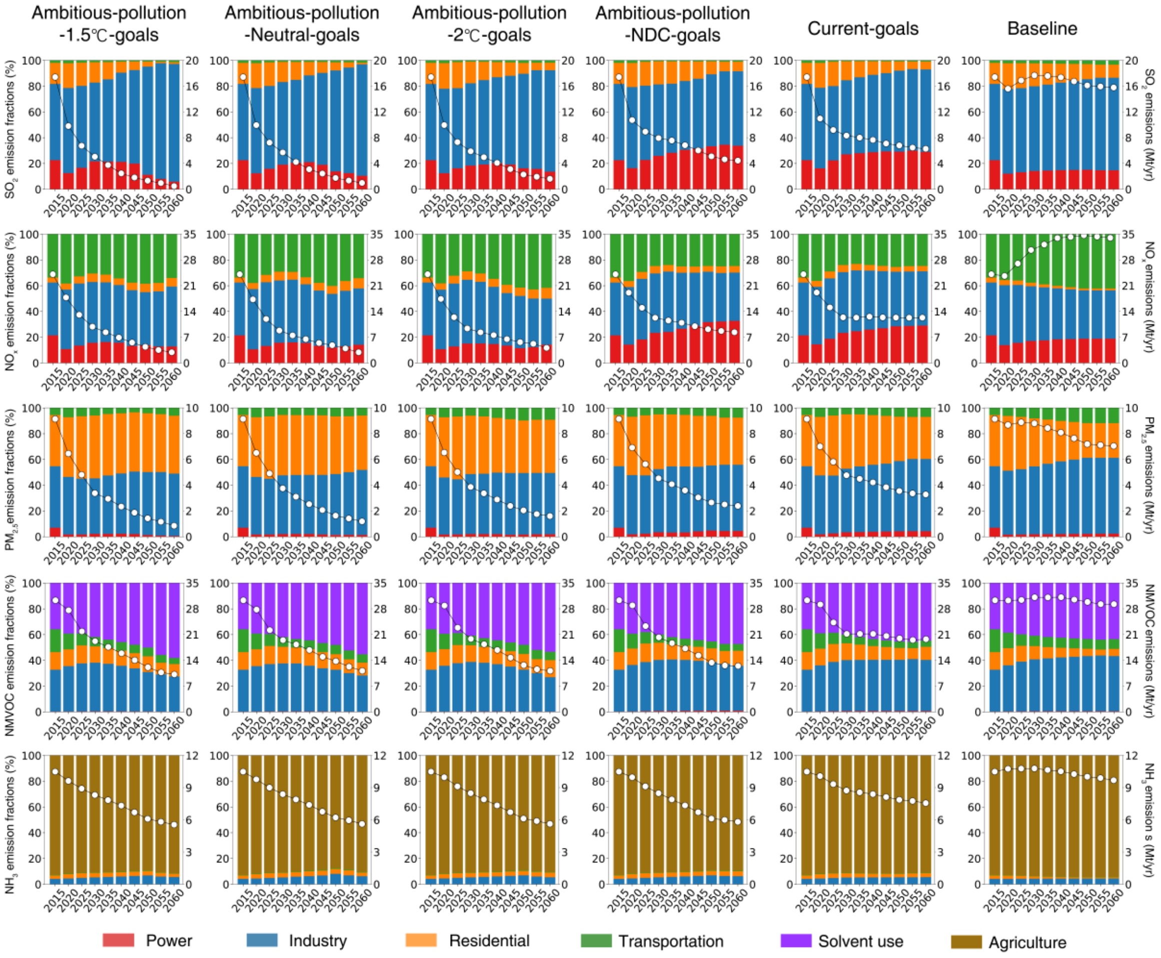

Fig. S3. Future anthropogenic emission (including SO_2, NO_x, $PM_{2.5}$, NMVOC, NH_3) pathways under different scenarios. The stacking histograms represent the emission fractions by sectors (left Y-axis), and the black lines with circle labels represent the emission magnitudes (right Y-axis).

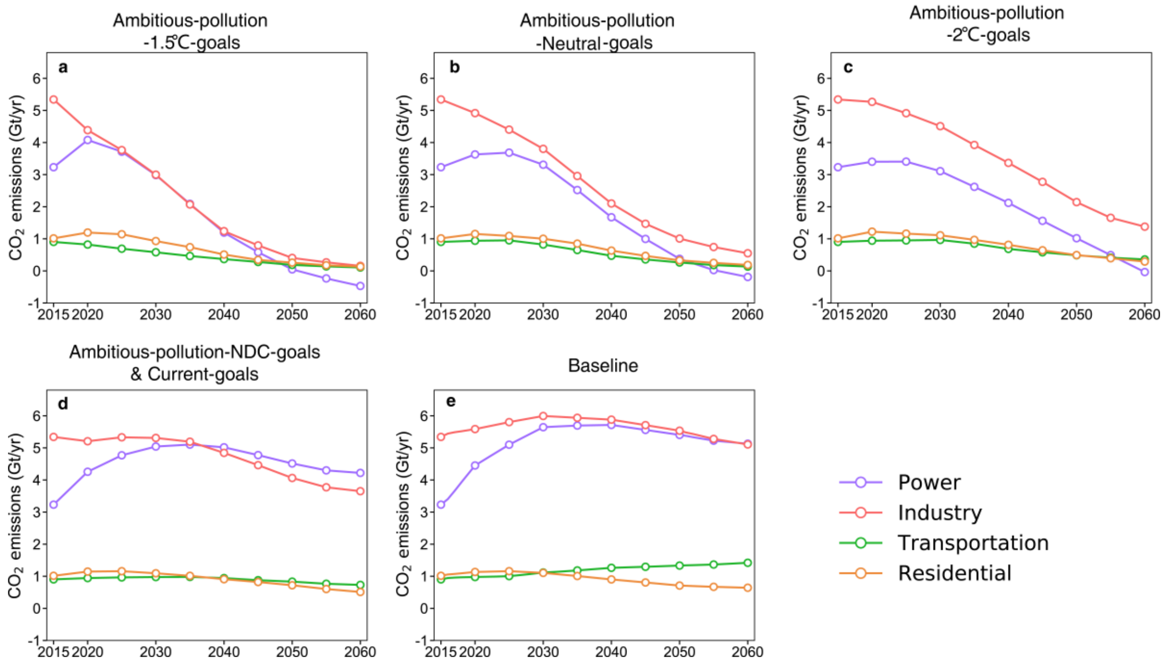

Fig. S4. Sectoral CO_2 emissions over China under different scenarios during 2015-2060.

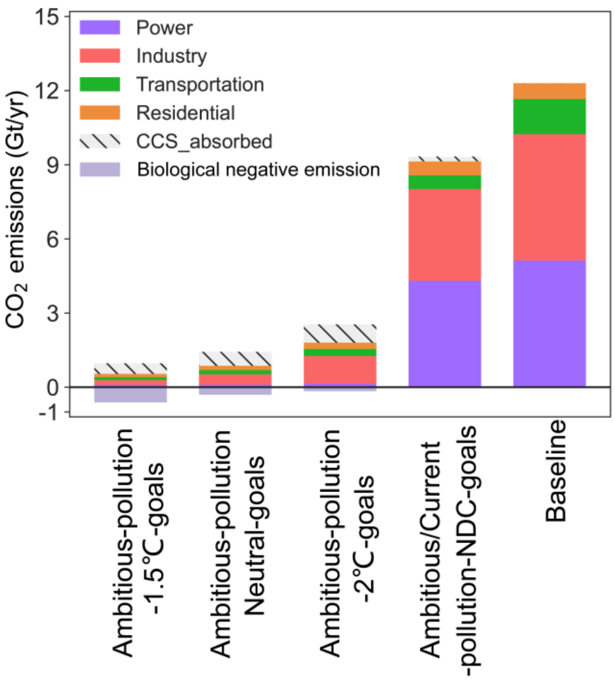

Fig. S5. Sectoral CO_2 emissions (including the positive CO_2 emissions from power, industry, transportation, residential sectors, absorbed positive CO_2 emissions by carbon capture and storage technologies, and negative CO_2 emissions by biological negative carbon emission technologies) over China under different scenarios in 2060.

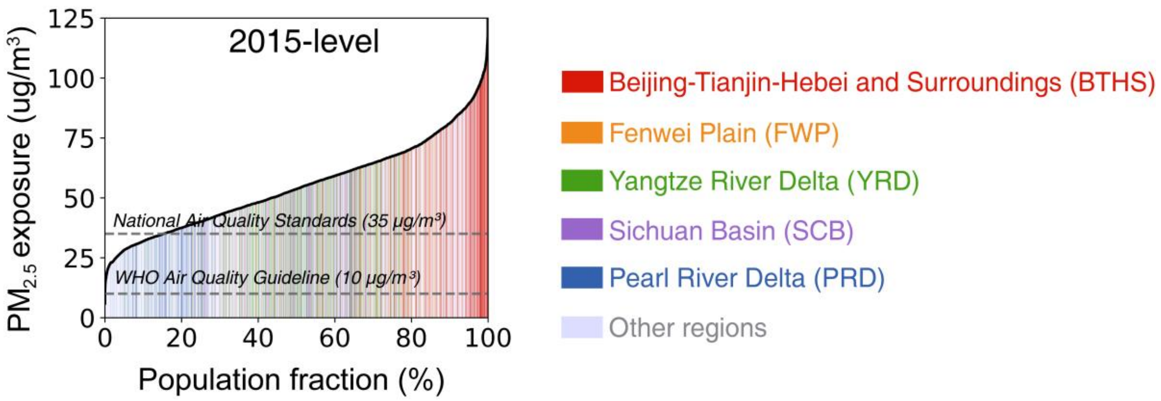

Fig. S6. Accumulated $PM_{2.5}$ exposure by 0.1° × 0.1° grid in 2015 (ranked from low to high, coloured by region). The horizontal grey dashed lines represent the national ambient air quality standards (i.e. 35 μg/m^3) and WHO Air Quality Guideline (i.e. 10 μg/m^3).

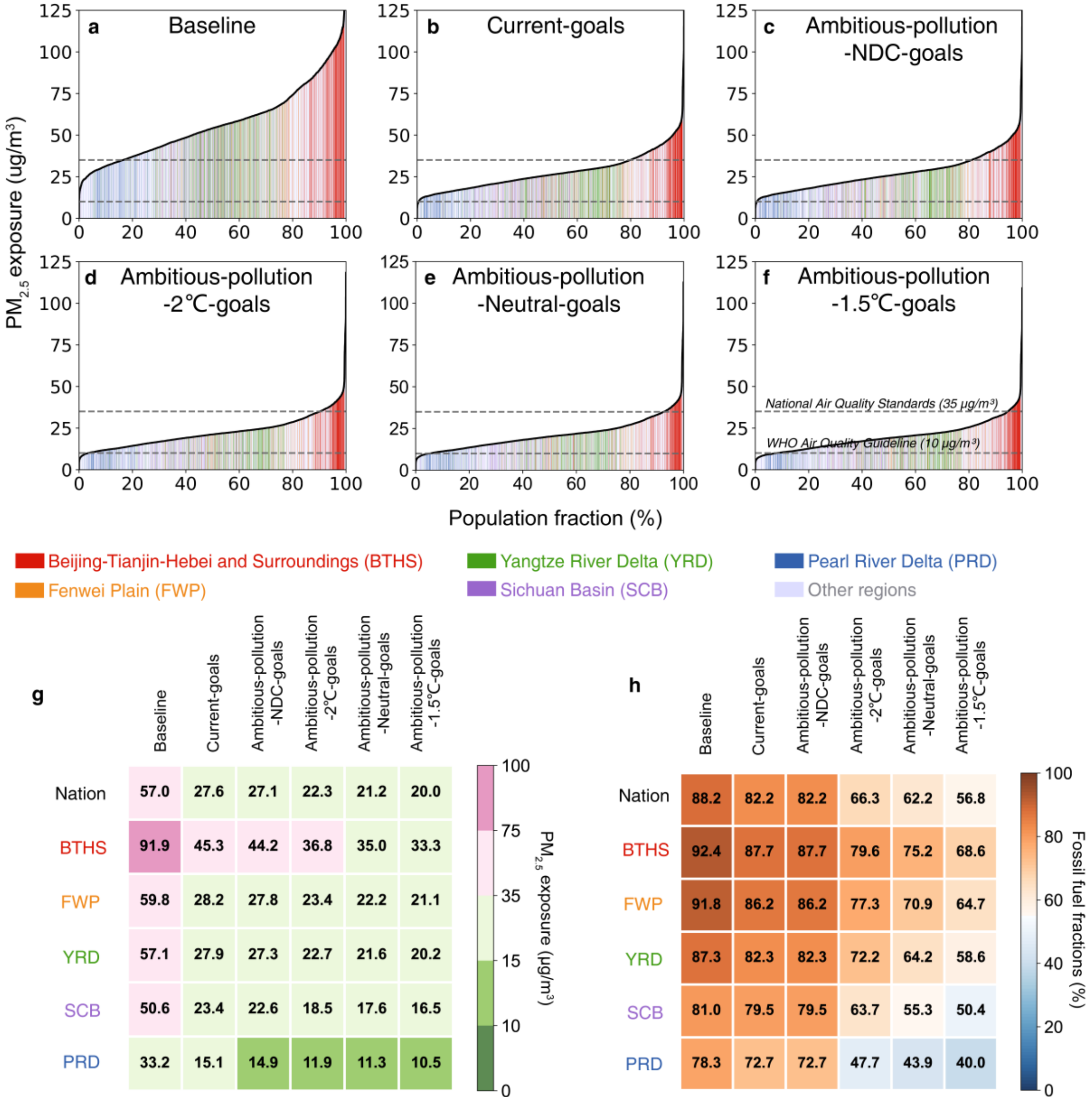

Fig. S7. Regional disparities of future $PM_{2.5}$ exposure and energy evolutions in 2030. (a)-(f), Accumulated $PM_{2.5}$ exposure by 0.1° × 0.1° grid in 2060 under different scenarios (ranked from low to high, coloured by region). The horizontal grey dashed lines represent the national ambient air quality standards (i.e. 35 μg/m^3) and WHO Air Quality Guideline (i.e. 10 μg/m^3). (g) National and regional population-weighted mean $PM_{2.5}$ concentrations in 2030. (h) Fossil fuel fraction in primary energy mix in 2030.

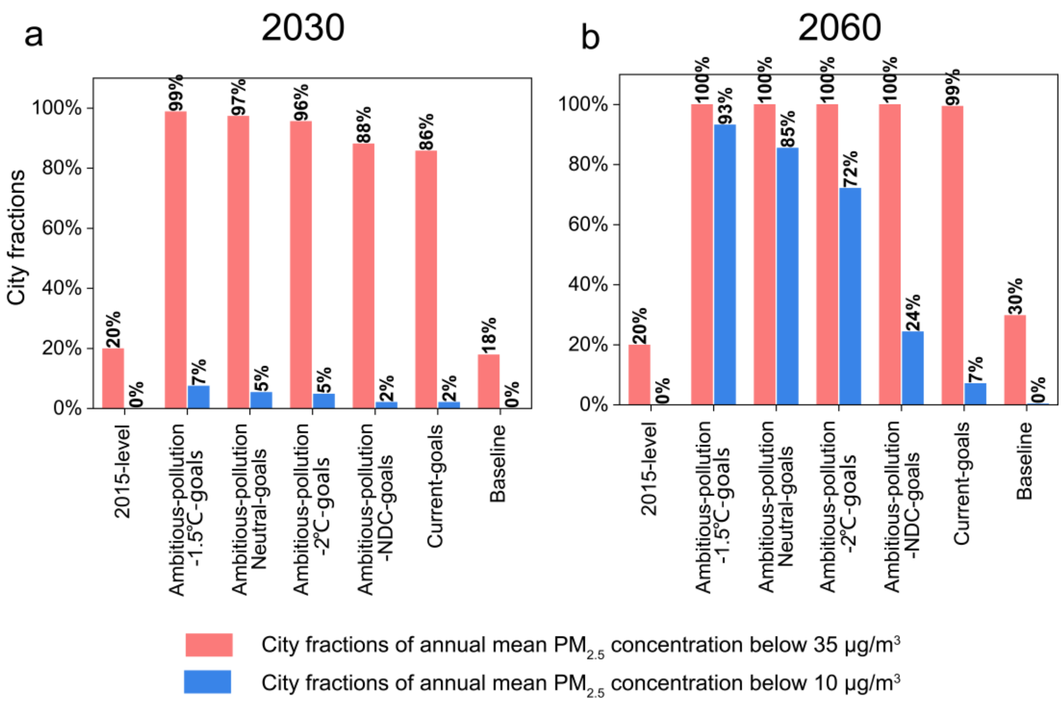

Fig. S8. City fractions of annual mean $PM_{2.5}$ concentrations below the national ambient air quality standards (i.e. 35 μg/m^3) and WHO Air Quality Guideline (i.e. 10 μg/m^3) in 2030 (a) and 2060 (b) under different scenarios.

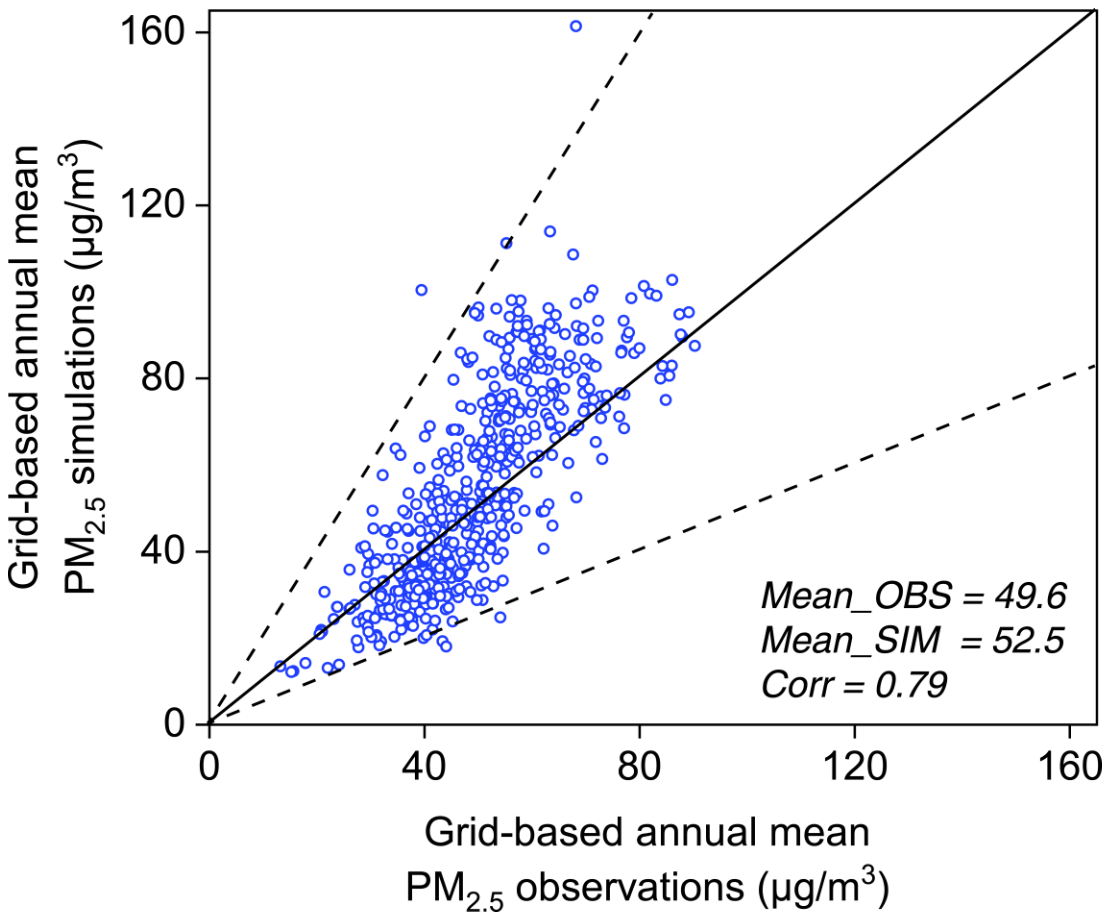

Fig. S9. Comparisons of the base-year (i.e. 2015) simulated and observed $PM_{2.5}$ concentrations (grid-based) over China. The solid line indicates the 1 : 1 line; while the dashed lines indicate the 1 : 2 and 2 : 1 lines.

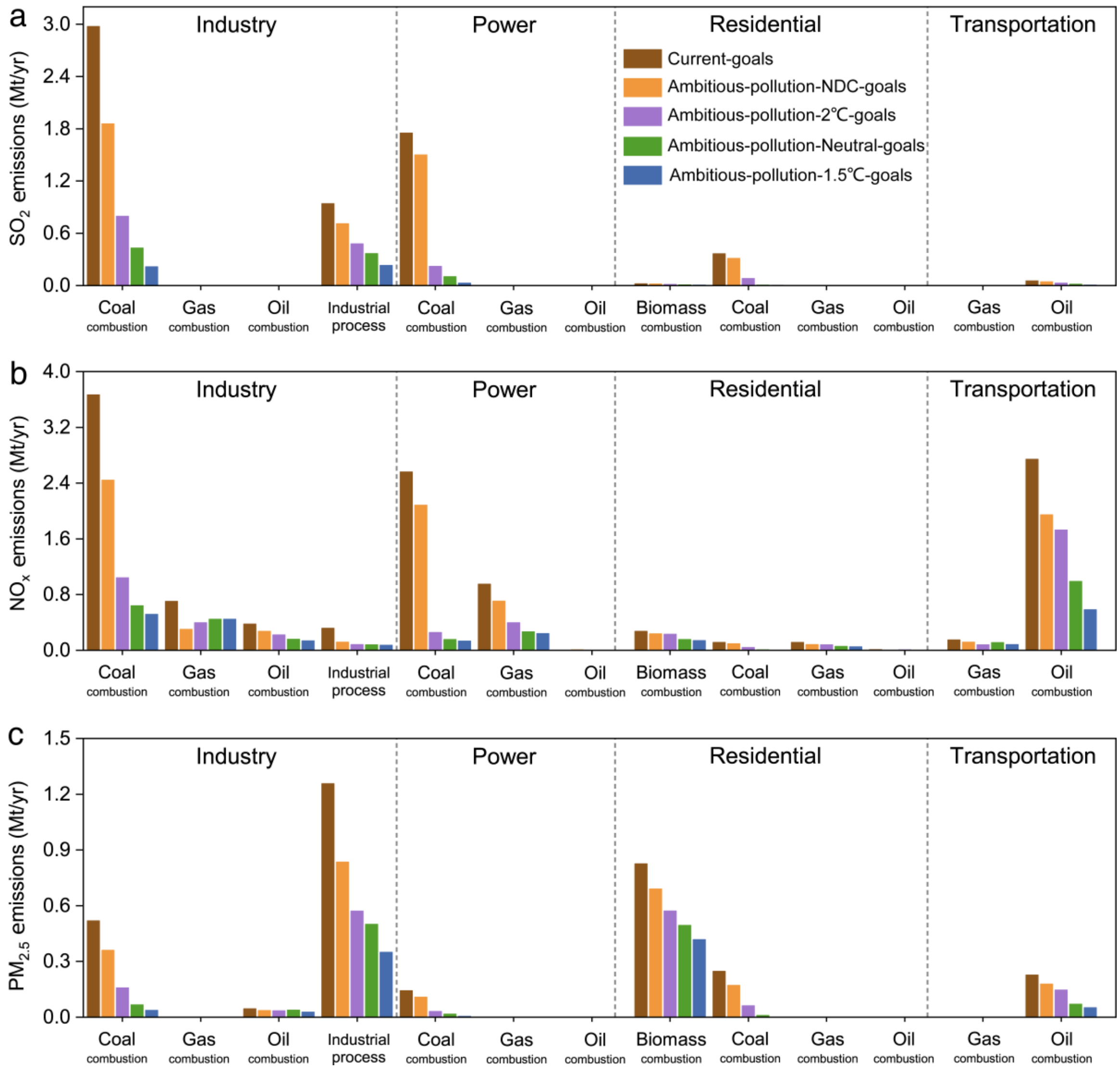

Fig. S10. China's major air pollutant emissions (SO_2, NO_x and primary $PM_{2.5}$) by sectors and fuel types in 2060 under different scenarios.

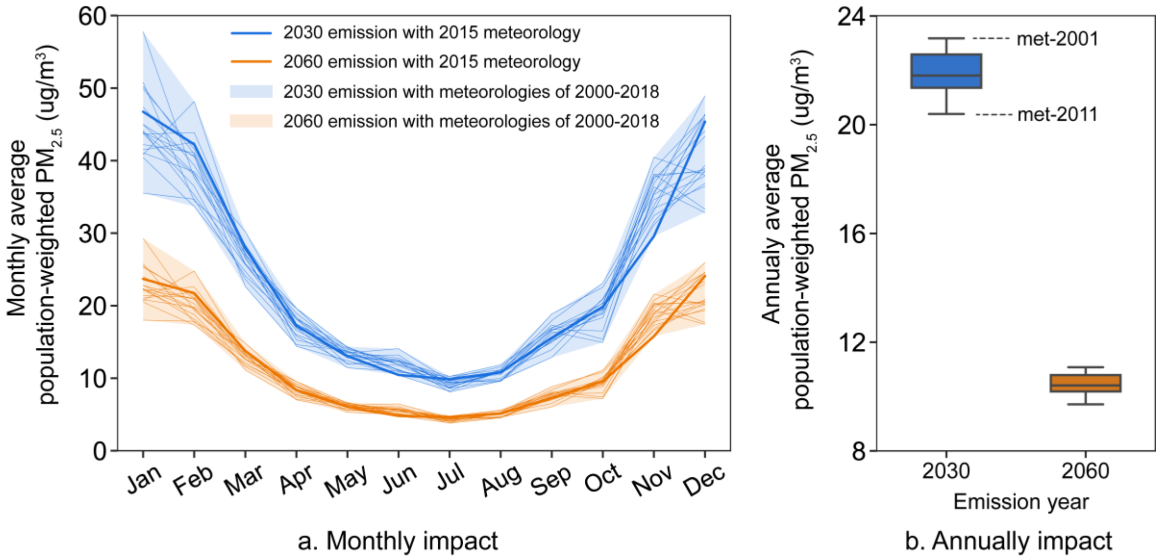

Fig. S11. Monthly (a) and annually (b) impacts of meteorology variations on China's future population-weighted mean $PM_{2.5}$ concentrations in 2030 and 2060 under the *Ambitious-pollution-2℃-goals* scenario.

Table S1.a. Detailed clean air measures, process, and policy parameters of the three air pollution control scenarios (i.e. *Baseline*, *Current-goals*, *Ambitious-pollution control*) in the power sector.

<table>
<tr><th>Emission source</th><th>Scenario</th><th>Region</th><th>Policy types</th><th>2015</th><th>2016</th><th>2017</th><th>2018</th><th>2019</th><th>2020</th><th>2021</th><th>…</th><th>2025</th><th>…</th><th>2028</th><th>2029</th><th>2030</th><th>2031</th><th>…</th><th>2035</th><th>…</th><th>2040</th><th>…</th><th>2045</th><th>2050</th><th>…</th><th>2060</th></tr>
<tr><td rowspan="10">Coal-fired power plants</td><td rowspan="2">Baseline</td><td rowspan="2">All region</td><td>End-of-pipe control</td><td colspan="23">GB 13223-2011 [SO_2-100; NO_x-100/200; PM-30]: end-of-pipe control remain the same as 2015</td></tr>
<tr><td>Power plant fleet turnover</td><td colspan="23">40-year lifetime by natural retirement</td></tr>
<tr><td rowspan="6">Current goals</td><td rowspan="2">BTHS & FWP</td><td>End-of-pipe control</td><td>GB 13223-2011</td><td colspan="5">achieving ultra-low standard[1] [SO_2-35; NO_x-50; PM-10]</td><td colspan="17">achieved ultra-low emission standard[1]: removal effeiciency of SO_2 >95%; NO_x >90%, de-PM devices update to at least FAB</td></tr>
<tr><td>Power plant fleet turnover</td><td colspan="6">phase out 15% of current installed capacity</td><td colspan="17">40-year lifetime by natural retirement</td></tr>
<tr><td rowspan="2">YRD</td><td>End-of-pipe control</td><td>GB 13223-2011</td><td colspan="3">achieving ultra-low standard[1]</td><td colspan="19">achieved ultra-low emission standard[1]</td></tr>
<tr><td>Power plant fleet turnover</td><td colspan="6">phase out 15% of current installed capacity</td><td colspan="17">40-year lifetime by natural retirement</td></tr>
<tr><td rowspan="2">Other regions</td><td>End-of-pipe control</td><td>GB 13223-2011</td><td colspan="5">achieving ultra-low standard[1]</td><td colspan="17">achieved ultra-low emission standard[1]</td></tr>
<tr><td>Power plant fleet turnover</td><td colspan="6">phase out 15% of current installed capacity</td><td colspan="17">40-year lifetime by natural retirement</td></tr>
<tr><td rowspan="2">Ambitious pollution control</td><td rowspan="2">All region</td><td>End-of-pipe control</td><td>GB 13223-2011</td><td colspan="3">achieving ultra-low standard[1]</td><td colspan="9">achieved ultra-low emission standard[1]</td><td colspan="2">achiveing BAT control[2]</td><td colspan="8">achieved BAT control[2] [SO_2-20; NO_x-30; PM-5]: removal effeiciency of SO_2 > 99%; NO_x > 95%, de-PM devices update to WESP</td></tr>
<tr><td>Power plant fleet turnover</td><td colspan="6">phase out 15% of current installed capacity</td><td colspan="17">further phase out 50% of current installed capacity</td></tr>
<tr><td rowspan="10">Other thermal power plants</td><td rowspan="2">Baseline</td><td rowspan="2">All region</td><td>End-of-pipe control</td><td colspan="23">GB 13223-2011: end-of-pipe control remain the same as 2015</td></tr>
<tr><td>Technology turnover</td><td colspan="13">GTCC technology increase to 10%</td><td colspan="10">-</td></tr>
<tr><td rowspan="6">Current goals</td><td rowspan="2">BTHS & FWP</td><td>End-of-pipe control</td><td colspan="3">GB 13223-2011</td><td colspan="3">achieving special limits[3] [SO2-50; NOx-100; PM-20]</td><td colspan="2">achieved special limits[3]: application rate of FGD > 80%, SCB,LNB > 70%</td><td colspan="2">achieving ultra-low emission[1]</td><td colspan="13">achieved ultra-low emission standard[1]: removal effeiciency of SO2 >95%; NOx >90%</td></tr>
<tr><td>Technology turnover</td><td colspan="8">GTCC technology increase to 30%; CFB technology increase to 40%</td><td colspan="15">GTCC technology increase to 50%; CFB technology increase to 60%</td></tr>
<tr><td rowspan="2">YRD</td><td>End-of-pipe control</td><td colspan="3">GB 13223-2011</td><td colspan="3">achieving special limits[3]</td><td colspan="2">achieved special limits[3]</td><td colspan="3">achieving ultra-low emission[1]</td><td colspan="12">achieved ultra-low emission standard[1]</td></tr>
<tr><td>Technology turnover</td><td colspan="8">GTCC technology increase to 30%; CFB technology increase to 40%</td><td colspan="15">GTCC technology increase to 50%; CFB technology increase to 60%</td></tr>
<tr><td rowspan="2">Other regions</td><td>End-of-pipe control</td><td colspan="3">GB 13223-2011</td><td colspan="3">achieving special limits[3]</td><td colspan="2">achieved special limits[3]</td><td colspan="6">achieving ultra-low emission[1]</td><td colspan="9">achieved ultra-low emission standard[1]</td></tr>
<tr><td>Technology turnover</td><td colspan="13">GTCC technology increase to 30%; CFB technology increase to 40%</td><td colspan="10">GTCC technology increase to 50%; CFB technology increase to 60%</td></tr>
<tr><td rowspan="2">Ambitious pollution control</td><td rowspan="2">All region</td><td>End-of-pipe control</td><td colspan="3">GB 13223-2011</td><td colspan="3">achieving special limits[3]</td><td colspan="2">achieved special limits[3]</td><td colspan="2">achieving ultra-low emission[1]</td><td colspan="6">achieved ultra-low emission standard[1]</td><td colspan="3">achiveing BAT control[2]</td><td colspan="4">achieved BAT control[2]</td></tr>
<tr><td>Technology turnover</td><td colspan="13">GTCC technology increase to 30%; CFB technology increase to 40%</td><td colspan="10">GTCC and CFB technology increase to 100%</td></tr>
</table>

Table S1.b. Detailed clean air measures, process, and policy parameters of the three air pollution control scenarios (i.e. *Baseline*, *Current-goals*, *Ambitious-pollution control*) in the industry sector.

<table>
<tr><th>Emission source</th><th>Scenario</th><th>Region</th><th>Policy types</th><th>2015</th><th>...</th><th>2018</th><th>2019</th><th>2020</th><th>2021</th><th>2022</th><th>2023</th><th>2024</th><th>2025</th><th>2026</th><th>2027</th><th>2028</th><th>2029</th><th>2030</th><th>...</th><th>2033</th><th>2034</th><th>2035</th><th>...</th><th>2040</th><th>...</th><th>2045</th><th>2046</th><th>2047</th><th>...</th><th>2050</th><th>...</th><th>2060</th></tr>
<tr><td rowspan="10">Coal-fired boilers (Heating and industrial)</td><td rowspan="2">Baseline</td><td rowspan="2">All region</td><td>End-of-pipe control</td><td colspan="29">GB 13271-2014 [SO_2-200/300; NO_x-200/300; PM-30/50]: end-of-pipe control remains the same as 2015</td></tr>
<tr><td>technology turnover</td><td colspan="15">phase out 10MW-below industry boilers in key regions</td><td colspan="14">-</td></tr>
<tr><td rowspan="6">Current goals</td><td rowspan="2">BTHS & FWP</td><td>End-of-pipe control</td><td colspan="2">GB 13271-2014</td><td colspan="2">achieving special limits[3]</td><td>achieved limits[3]</td><td colspan="3">achieving ultra-low standard[1]</td><td colspan="21">achieved ultra-low emission standard[1]</td></tr>
<tr><td>technology turnover</td><td colspan="2">phase out 10MW-below industry boilers</td><td colspan="3">phase out 25MW-below industry boilers</td><td colspan="10">phase out 35MW-below industry boilers</td><td colspan="14">-</td></tr>
<tr><td rowspan="2">YRD</td><td>End-of-pipe control</td><td colspan="2">GB 13271-2014</td><td colspan="2">achieving limits[3]</td><td>achieved limits[3]</td><td colspan="4">achieving ultra-low standard[1]</td><td colspan="20">achieved ultra-low emission standard[1]</td></tr>
<tr><td>technology turnover</td><td colspan="2">phase out 10MW-below industry boilers</td><td colspan="6">phase out 25MW-below industry boilers</td><td colspan="7">phase out 35MW-below industry boilers</td><td colspan="14">-</td></tr>
<tr><td rowspan="2">Other regions</td><td>End-of-pipe control</td><td colspan="2">GB 13271-2014</td><td colspan="2">achieving limits[3]</td><td>achieved limits[3]</td><td colspan="5">achieving ultra-low standard[1]</td><td colspan="19">achieved ultra-low emission standard[1]</td></tr>
<tr><td>technology turnover</td><td colspan="2">phase out 10MW-below industry boilers</td><td colspan="13">phase out 25MW-below industry boilers</td><td colspan="14">-</td></tr>
<tr><td rowspan="2">Ambitious pollution control</td><td rowspan="2">All region</td><td>End-of-pipe control</td><td colspan="2">GB 13271-2014</td><td colspan="2">achieving limits[3]</td><td>achieved limits[3]</td><td colspan="3">achieving ultra-low standard[1]</td><td colspan="10">achieved ultra-low emission standard[1]</td><td colspan="4">achiveing BAT control[2]</td><td colspan="7">achieved BAT control[2]</td></tr>
<tr><td>technology turnover</td><td colspan="2">phase out 10MW-below industry boilers</td><td colspan="3">phase out 25MW-below industry boilers</td><td colspan="10">phase out 35MW-below industry boilers</td><td colspan="12">phase out all the outdated capacities</td><td colspan="2">-</td></tr>
<tr><td rowspan="10">Iron and steel plants (Sinter/Coke/Iron/Steel)</td><td rowspan="2">Baseline</td><td rowspan="2">All region</td><td>End-of-pipe control</td><td colspan="29">GB 28662-2012 (sinter) [SO_2-200; NO_x-300; PM-50] / GB16171-2012 (coke) [SO_2-100; NO_x-200; PM-50] / GB 28663-2012 (iron) [PM-20; PM-fu-8] / GB 28664-2012 (steel) [PM-20/50; PM-fu-8]: end-of-pipe control remains the same as 2015</td></tr>
<tr><td>technology turnover</td><td colspan="29">-</td></tr>
<tr><td rowspan="6">Current goals</td><td rowspan="2">BTHS & FWP</td><td>End-of-pipe control</td><td colspan="2">standards</td><td colspan="2">special limits[4] [SO_2-50/100; NO_x-100/320; PM-15/20]</td><td colspan="6">achieving ultra-low emission standard[5] [SO_2-35/50; NO_x-50/200; PM-10]</td><td colspan="19">achieved ultra-low emission standard[5] : removal effeiciency of SO_2 ~90%-95%; NO_x ~80%-90%, de-PM devices update to at least FAB, improve fugitive dust control by 20%</td></tr>
<tr><td>technology turnover</td><td colspan="2">phase out old iron/steel/coke capacities by 215 million tonnes</td><td colspan="3">phase out old iron/steel/ coke capacities by 100 million tonnes</td><td colspan="5">further phase out outdated iron/steel/coke capacities by 20%</td><td colspan="19">-</td></tr>
<tr><td rowspan="2">YRD</td><td>End-of-pipe control</td><td colspan="2">standards</td><td colspan="2">special limits[4]</td><td colspan="6">achieving ultra-low emission standard[5]</td><td colspan="19">achieved ultra-low emission standard[5]</td></tr>
<tr><td>technology turnover</td><td colspan="2">phase out old iron/steel/coke capacities by 30 million tonnes</td><td colspan="3">phase out old iron/steel/ coke capacities by 10 million tonnes</td><td colspan="5">further phase out outdated iron/steel/coke capacities by 20%</td><td colspan="19">-</td></tr>
<tr><td rowspan="2">Other regions</td><td>End-of-pipe control</td><td colspan="2">standards</td><td colspan="3">special limits[4]</td><td colspan="10">achieving ultra-low emission standard[5]</td><td colspan="14">achieved ultra-low emission standard[5]</td></tr>
<tr><td>technology turnover</td><td colspan="2">phase out old iron/steel/coke capacities by 3 million tonnes</td><td colspan="3">phase out old iron/steel/ coke capacities by 60 million tonnes</td><td colspan="10">further phase out outdated iron/steel/coke capacities by 20%</td><td colspan="14">-</td></tr>
<tr><td rowspan="2">Ambitious pollution control</td><td rowspan="2">All region</td><td>End-of-pipe control</td><td colspan="2">standards</td><td colspan="2">special limits[4]</td><td colspan="6">achieving ultra-low emission standard[5]</td><td colspan="7">achieved ultra-low emission standard[5]</td><td colspan="5">achieving BAT control[6] [SO_2 35; NO_x-50/100; PM-10]</td><td colspan="7">achieved BAT control[6]: removal effeiciency of SO2 >98%; NOx >93%, de-PM devices update to WESP</td></tr>
<tr><td>technology turnover</td><td colspan="2">phase out old iron/steel/coke capacities by 250 million tonnes</td><td colspan="3">phase out old iron/steel/ coke capacities by 170 million tonnes</td><td colspan="5">further phase out outdated iron/steel/coke capacities by 20%</td><td colspan="17">phase out all the iron/steel/coke outdated capacities</td><td colspan="2">-</td></tr>
</table>

Emission source	Scenario	Region	Policy types	2015	...	2018	2019	2020	2021	2022	2023	2024	2025	2026	2027	2028	2029	2030	...	2033	2034	2035	...	2040	...	2045	2046	2047	...	2050	...	2060
Nonferrous metal	Baseline	All region	End-of-pipe control	GB 31574-201529(Pb,Zn), GB 31575-201530(Cu,Al), GB 25468-201031(Mg), GB 25467-201032(Ni) [SO_2-150/200; NO_x-200; PM-30/50]: end-of-pipe control remains the same as 2015																												
			technology turnover	-																												
	Current goals	BTHS & FWP	End-of-pipe control	standards		achieving special limits[7]		achieved special limits[7] [SO_2-100; NO_x-100; PM-15/20]					achieving ultra-low emission standard[5]			achieved ultra-low emission standard[5]																
			technology turnover	-		phase out old nonferrous metal capacities by 10%			further phase out outdated nonferrous metal capacities by 20%										-													
		YRD	End-of-pipe control	standards		achieving special limits[7]		achieved special limits[7]					achieving ultra-low emission standard[5]				achieved ultra-low emission standard[5]															
			technology turnover	-		phase out old nonferrous metal capacities by 10%			further phase out outdated nonferrous metal capacities by 20%										-													
		Other regions	End-of-pipe control	standards		achieving special limits[7]			achieved special limits[7]				achieving ultra-low emission standard[5]						achieved ultra-low emission standard[5]													
			technology turnover	-		phase out old nonferrous metal capacities by 10%						further phase out outdated nonferrous metal capacities by 20%							-													
	Ambitious pollution control	All region	End-of-pipe control	standards		achieving special limits[7]		achieved special limits[7]					achieving ultra-low emission standard[5]			achieved ultra-low emission standard[5]								achieving BAT control[6]					achieved BAT control[6]			
			technology turnover	-		phase out old nonferrous metal capacities by 10%			further phase out outdated nonferrous metal capacities by 20%										phase out all the nonferrous metal outdated capacities													-
Cement plants	Baseline	All region	End-of-pipe control	GB 4915-2013 [SO_2-200; NO_x-400; PM-30]: end-of-pipe control remains the same as 2015																												
			cement klin turnover	Cement energy norm					-																							
	Current goals	BTHS & FWP	End-of-pipe control	GB 4915-2013		achieving special limits[8]		achieved special limits[8] [SO_2-100; NO_x-320; PM-20]			achieving ultra-low emission standard[9] [SO_2-50; NO_x-100; PM-15]			achieved ultra-low emission standard[9]: removal effeiciency of SO_2 ~90%-95%; NO_x ~80%-90%, de-PM devices update to at least FAB, improve fugitive dust control by 20%																		
			cement klin turnover	phase out old cement capacities by 100 million tonnes		phase out old cement capacities by 50 million tonnes			further phase out outdated cement capacities by 20%					-																		
		YRD	End-of-pipe control	GB 4915-2013		achieving special limits[8]		achieved special limits[8]			achieving ultra-low emission standard[9]				achieved ultra-low emission standard[9]																	
			cement klin turnover	phase out old cement capacities by 32 million tonnes		phase out old cement capacities by 10 million tonnes			further phase out outdated cement capacities by 20%					-																		
		Other regions	End-of-pipe control	GB 4915-2013		achieving special limits[8]			special limits[8]		achieving ultra-low emission standard[9]								achieved ultra-low emission standard[9]													
			cement klin turnover	phase out old cement capacities by 120 million tonnes		phase out old cement capacities by 130 million tonnes			further phase out outdated cement capacities by 20%										-													
	Ambitious pollution control	All region	End-of-pipe control	GB 4915-2013		achieving special limits[8]		achieved special limits[8]			achieving ultra-low emission standard[9]			achieved ultra-low emission standard[9]										achieving BAT control[10] [SO_2-35; NO_x-50; PM-10]			achieved BAT control[10]: removal effeiciency of SO_2 > 98%; NOx > 95%, de-PM devices update to WESP					
			cement klin turnover	phase out old cement capacities by 252 million tonnes		phase out old cement capacities by 190 million tonnes			further phase out outdated cement capacities by 20%					phase out all the outdated cement capacities; penetration rate of klin with capacities over 4000 tons > 70%																	-	

<table>
<tr><th>Emission source</th><th>Scenario</th><th>Region</th><th>Policy types</th><th>2015</th><th>...</th><th>2018</th><th>2019</th><th>2020</th><th>2021</th><th>2022</th><th>2023</th><th>2024</th><th>2025</th><th>2026</th><th>2027</th><th>2028</th><th>2029</th><th>2030</th><th>...</th><th>2033</th><th>2034</th><th>2035</th><th>...</th><th>2040</th><th>...</th><th>2045</th><th>2046</th><th>2047</th><th>...</th><th>2050</th><th>...</th><th>2060</th></tr>
<tr><td rowspan="10">Flat glass</td><td rowspan="2">Baseline</td><td rowspan="2">All region</td><td>End-of-pipe control</td><td colspan="29">GB29495-2013 [SO_2-400; NO_x-700; PM-50]: end-of-pipe control remains the same as 2015</td></tr>
<tr><td>technology turnover</td><td colspan="29">-</td></tr>
<tr><td rowspan="6">Current goals</td><td rowspan="2">BTHS & FWP</td><td>End-of-pipe control</td><td colspan="2">GB29495-2013</td><td colspan="2">achieving special limits[11]</td><td colspan="5">achieved special limits[11] [SO_2-200; NO_x-300; PM-30]</td><td colspan="3">achieving ultra-low emission standard[9]</td><td colspan="17">achieved ultra-low emission standard[9]</td></tr>
<tr><td>technology turnover</td><td colspan="2">phase out old flat glass capacities by 78 million weight cases</td><td colspan="3">phase out old flat glass capacities by 8 million weight cases</td><td colspan="5">further phase out outdated flat glass capacities by 20%</td><td colspan="19">-</td></tr>
<tr><td rowspan="2">YRD</td><td>End-of-pipe control</td><td colspan="2">GB29495-2013</td><td colspan="2">achieving special limits[11]</td><td colspan="5">achieved special limits[11]</td><td colspan="4">achieving ultra-low emission standard[9]</td><td colspan="16">achieved ultra-low emission standard[9]</td></tr>
<tr><td>technology turnover</td><td colspan="2">phase out old flat glass capacities by 11 million weight cases</td><td colspan="8">further phase out outdated flat glass capacities by 20%</td><td colspan="19">-</td></tr>
<tr><td rowspan="2">Other regions</td><td>End-of-pipe control</td><td colspan="2">GB29495-2013</td><td colspan="3">achieving special limits[11]</td><td colspan="4">achieved special limits[11]</td><td colspan="6"></td><td colspan="14">achieved ultra-low emission standard[9]</td></tr>
<tr><td>technology turnover</td><td colspan="2">phase out old flat glass capacities by 22 million weight cases</td><td colspan="3">phase out old flat glass capacities by 6 million weight cases</td><td colspan="10">further phase out outdated flat glass capacities by 20%</td><td colspan="14">-</td></tr>
<tr><td rowspan="2">Ambitious pollution control</td><td rowspan="2">All region</td><td>End-of-pipe control</td><td colspan="2">GB29495-2013</td><td colspan="2">achieving special limits[11]</td><td colspan="5">achieved special limits[11]</td><td colspan="3">achieving ultra-low emission standard[9]</td><td colspan="9">achieved ultra-low emission standard[9]</td><td colspan="3">achieving BAT control[10]</td><td colspan="5">achieved BAT control[10]</td></tr>
<tr><td>technology turnover</td><td colspan="2">phase out old flat glass capacities by 111 million weight cases</td><td colspan="3">phase out old flat glass capacities by 14 million weight cases</td><td colspan="5">further phase out outdated flat glass capacities by 20%</td><td colspan="17">phase out all the outdated flat glass capacities</td><td colspan="2">-</td></tr>
<tr><td rowspan="10">Brick/lime and other indutires</td><td rowspan="2">Baseline</td><td rowspan="2">All region</td><td>End-of-pipe control</td><td colspan="29">GB 29620-2013 (brick and lime) [SO_2-300; NO_x-200; PM-30]: end-of-pipe control remains the same as 2015</td></tr>
<tr><td>technology turnover</td><td colspan="29">-</td></tr>
<tr><td rowspan="6">Current goals</td><td rowspan="2">BTHS & FWP</td><td>End-of-pipe control</td><td colspan="2">standards</td><td colspan="2">achieving special limits[8]</td><td colspan="5">achieved special limits[8]</td><td colspan="4">achieving ultra-low emission standard[9]</td><td colspan="16">achieved ultra-low emission standard[9]</td></tr>
<tr><td>technology turnover</td><td colspan="2">-</td><td colspan="3">phase out old brick/lime capacities by 10%</td><td colspan="10">further phase out outdated brick/lime capacities by 20%</td><td colspan="14">-</td></tr>
<tr><td rowspan="2">YRD</td><td>End-of-pipe control</td><td colspan="2">standards</td><td colspan="2">achieving special limits[8]</td><td colspan="5">achieved special limits[8]</td><td colspan="5">achieving ultra-low emission standard[9]</td><td colspan="15">achieved ultra-low emission standard[9]</td></tr>
<tr><td>technology turnover</td><td colspan="2">-</td><td colspan="3">phase out old brick/lime capacities by 10%</td><td colspan="10">further phase out outdated brick/lime capacities by 20%</td><td colspan="14">-</td></tr>
<tr><td rowspan="2">Other regions</td><td>End-of-pipe control</td><td colspan="2">standards</td><td colspan="3">achieving special limits[8]</td><td colspan="4">achieved special limits[8]</td><td colspan="6">achieving ultra-low emission standard[9]</td><td colspan="14">achieved ultra-low emission standard[9]</td></tr>
<tr><td>technology turnover</td><td colspan="2">-</td><td colspan="6">phase out old brick/lime capacities by 10%</td><td colspan="7">further phase out outdated brick/lime capacities by 20%</td><td colspan="14">-</td></tr>
<tr><td rowspan="2">Ambitious pollution control</td><td rowspan="2">All region</td><td>End-of-pipe control</td><td colspan="2">standards</td><td colspan="2">achieving special limits[8]</td><td colspan="5">achieved special limits[8]</td><td colspan="4">achieving ultra-low emission standard[9]</td><td colspan="8">achieved ultra-low emission standard[9]</td><td colspan="4">achieving BAT control[10]</td><td colspan="4">achieved BAT control[10]</td></tr>
<tr><td>technology turnover</td><td colspan="2">-</td><td colspan="3">phase out old brick/lime capacities by 10%</td><td colspan="10">further phase out outdated brick/lime capacities by 20%</td><td colspan="12">phase out all the outdated brick/lime capacities</td><td colspan="2">-</td></tr>
<tr><td rowspan="5">Key VOCs-related indutiries</td><td>Baseline</td><td>All region</td><td>End-of-pipe control</td><td colspan="29">-</td></tr>
<tr><td rowspan="3">Current goals</td><td>BTHS & FWP</td><td>End-of-pipe control</td><td colspan="2">-</td><td colspan="3">improve LDAR</td><td colspan="7">further improve LDAR technology; install VOCs control facility in all VOC-related industries</td><td colspan="17">relative low emission levels</td></tr>
<tr><td>YRD</td><td>End-of-pipe control</td><td colspan="2">-</td><td colspan="3">improve LDAR</td><td colspan="8">further improve the LDAR technology; install VOC control facility in all VOC-related industries</td><td colspan="16">relative low emission levels</td></tr>
<tr><td>Other regions</td><td>End-of-pipe control</td><td colspan="2">-</td><td colspan="6">improve LDAR in chemical industry</td><td colspan="7">further improve the LDAR technology; install VOC control facility in all VOC-related industries</td><td colspan="14">relative low emission levels</td></tr>
<tr><td>Ambitious pollution</td><td>All region</td><td>End-of-pipe control</td><td colspan="2">-</td><td colspan="3">improve LDAR</td><td colspan="7">further improve LDAR technology; install VOCs control facility in all VOC-related industries</td><td colspan="10">relative low emission levels</td><td colspan="7">Innovation of VOC control facilities</td></tr>
</table>

Table S1.c. Detailed clean air measures, process, and policy parameters of the three air pollution control scenarios (i.e. *Baseline*, *Current-goals*, *Ambitious-pollution control*) in the transportation sector.

<table>
<tr><th>Emission source</th><th>Scenario</th><th>Region</th><th>Policy types</th><th>2015</th><th>2016</th><th>2017</th><th>2018</th><th>2019</th><th>2020</th><th>2021</th><th>2022</th><th>2023</th><th>2024</th><th>2025</th><th>…</th><th>2030</th><th>…</th><th>2034</th><th>2035</th><th>…</th><th>2040</th><th>…</th><th>2046</th><th>…</th><th>2050</th><th>…</th><th>2060</th></tr>
<tr><td rowspan="5">Light-duty gasoline vehicle</td><td>Baseline</td><td>All region</td><td>End-of-pipe control</td><td colspan="2">China 4</td><td colspan="22">China 5</td></tr>
<tr><td rowspan="3">Current goals</td><td>BTHS & FWP</td><td>End-of-pipe control</td><td colspan="2">China 4</td><td colspan="2">China 5</td><td colspan="3">China 6a</td><td colspan="17">China 6b</td></tr>
<tr><td>YRD</td><td>End-of-pipe control</td><td colspan="2">China 4</td><td colspan="2">China 5</td><td colspan="3">China 6a</td><td colspan="17">China 6b</td></tr>
<tr><td>Other regions</td><td>End-of-pipe control</td><td colspan="2">China 4</td><td colspan="3">China 5</td><td colspan="3">China 6a</td><td colspan="16">China 6b</td></tr>
<tr><td>Ambitious pollution control</td><td>All region</td><td>End-of-pipe control</td><td colspan="2">China 4</td><td colspan="2">China 5</td><td colspan="3">China 6a</td><td colspan="6">China 6b</td><td colspan="11">Assumed China 7</td></tr>
<tr><td rowspan="5">Heavy-duty gasoline vehicle</td><td>Baseline</td><td>All region</td><td>End-of-pipe control</td><td colspan="24">China 4</td></tr>
<tr><td rowspan="3">Current goals</td><td>BTHS & FWP</td><td>End-of-pipe control</td><td colspan="5">China 4</td><td colspan="2">China 5</td><td colspan="3">China 6a</td><td colspan="14">China 6b</td></tr>
<tr><td>YRD</td><td>End-of-pipe control</td><td colspan="5">China 4</td><td colspan="2">China 5</td><td colspan="3">China 6a</td><td colspan="14">China 6b</td></tr>
<tr><td>Other regions</td><td>End-of-pipe control</td><td colspan="6">China 4</td><td colspan="2">China 5</td><td colspan="3">China 6a</td><td colspan="13">China 6b</td></tr>
<tr><td>Ambitious pollution control</td><td>All region</td><td>End-of-pipe control</td><td colspan="5">China 4</td><td colspan="2">China 5</td><td colspan="3">China 6a</td><td colspan="6">China 6b</td><td colspan="8">Assumed China 7</td></tr>
<tr><td rowspan="5">Light-duty diesel vehicle</td><td>Baseline</td><td>All region</td><td>End-of-pipe control</td><td colspan="3">China IV</td><td colspan="21">China V</td></tr>
<tr><td rowspan="3">Current goals</td><td>BTHS & FWP</td><td>End-of-pipe control</td><td colspan="3">China IV</td><td colspan="4">China V</td><td colspan="2">China VI a</td><td colspan="15">China VI b</td></tr>
<tr><td>YRD</td><td>End-of-pipe control</td><td colspan="3">China IV</td><td colspan="4">China V</td><td colspan="2">China VI a</td><td colspan="15">China VI b</td></tr>
<tr><td>Other regions</td><td>End-of-pipe control</td><td colspan="3">China IV</td><td colspan="4">China V</td><td colspan="2">China VI a</td><td colspan="15">China VI b</td></tr>
<tr><td>Ambitious pollution control</td><td>All region</td><td>End-of-pipe control</td><td colspan="3">China IV</td><td colspan="4">China V</td><td colspan="2">China VI a</td><td colspan="6">China VI b</td><td colspan="9">Assumed China VII</td></tr>
<tr><td rowspan="5">Heavy-duty diesel vehicle</td><td>Baseline</td><td>All region</td><td>End-of-pipe control</td><td colspan="2">China IV</td><td colspan="22">China 5</td></tr>
<tr><td rowspan="3">Current goals</td><td>BTHS & FWP</td><td>End-of-pipe control</td><td colspan="2">China IV</td><td colspan="4">China V</td><td colspan="2">China VI a</td><td colspan="16">China VI b</td></tr>
<tr><td>YRD</td><td>End-of-pipe control</td><td colspan="2">China IV</td><td colspan="4">China V</td><td colspan="2">China VI a</td><td colspan="16">China VI b</td></tr>
<tr><td>Other regions</td><td>End-of-pipe control</td><td colspan="2">China IV</td><td colspan="4">China V</td><td colspan="2">China VI a</td><td colspan="16">China VI b</td></tr>
<tr><td>Ambitious pollution control</td><td>All region</td><td>End-of-pipe control</td><td colspan="2">China IV</td><td colspan="4">China V</td><td colspan="2">China VI a</td><td colspan="10">China VI b</td><td colspan="6">Assumed China VII</td></tr>
<tr><td rowspan="5">All types of on-road vehicle</td><td>Baseline</td><td>All region</td><td>Mobile fleet turnover</td><td colspan="3">phase out 20 million old vehicles</td><td colspan="21">-</td></tr>
<tr><td rowspan="3">Current goals</td><td>BTHS & FWP</td><td>Mobile fleet turnover</td><td colspan="3">phase out 8 million old vehicles</td><td colspan="3">phase out 2 million old vehicles</td><td colspan="7">phase out all the old vehicles</td><td colspan="11">-</td></tr>
<tr><td>YRD</td><td>Mobile fleet turnover</td><td colspan="3">phase out 2.6 million old vehicles</td><td colspan="3">phase out 0.5 million old vehicles</td><td colspan="7">phase out all the old vehicles</td><td colspan="11">-</td></tr>
<tr><td>Other regions</td><td>Mobile fleet turnover</td><td colspan="3">phase out 9.4 million old vehicles</td><td colspan="3">phase out 3 million old vehicles</td><td colspan="18">-</td></tr>
<tr><td>Ambitious pollution control</td><td>All region</td><td>Mobile fleet turnover</td><td colspan="3">phase out 20 million old vehicles</td><td colspan="3">phase out 5.5 million old vehicles</td><td colspan="7">phase out all the old vehicles</td><td colspan="11">-</td></tr>
<tr><td rowspan="5">Off-road</td><td>Baseline</td><td>All region</td><td>End-of-pipe control</td><td>China II</td><td colspan="23">China III</td></tr>
<tr><td rowspan="3">Current goals</td><td>BTHS & FWP</td><td>End-of-pipe control</td><td>China II</td><td colspan="4">China III</td><td colspan="4">China IV</td><td colspan="15">China V</td></tr>
<tr><td>YRD</td><td>End-of-pipe control</td><td>China II</td><td colspan="4">China III</td><td colspan="4">China IV</td><td colspan="15">China V</td></tr>
<tr><td>Other regions</td><td>End-of-pipe control</td><td>China II</td><td colspan="4">China III</td><td colspan="5">China IV</td><td colspan="14">China V</td></tr>
<tr><td>Ambitious pollution control</td><td>All region</td><td>End-of-pipe control</td><td>China II</td><td colspan="4">China III</td><td colspan="4">China IV</td><td colspan="8">China V</td><td colspan="3">China VI a</td><td colspan="4">China VI b</td></tr>
</table>

Table S1.d. Detailed clean air measures, process, and policy parameters of the three air pollution control scenarios (i.e. *Baseline*, *Current-goals*, *Ambitious-pollution control*) in the residential, agriculture and solvent use sectors.

Source sector	Scenario	Region	2015	2016	2017	2018	2019	2020	2021	…	2026	2027	2028	…	2030	…	2045	…	2050	…	2060
Residential	Baseline	All region	-																		
	Current goals	BTHS & FWP	-			cleaner coals and stoves			further reduce the ash and sulfur content in residential coal, and fully applied advanced stoves			relative low emission levels									
		YRD	-			cleaner coals and stoves			further reduce the ash and sulfur content in residential coal, and fully applied advanced stoves					relative low emission levels							
		Other regions	-			cleaner coals and stoves			further reduce the ash and sulfur content in residential coal, and fully applied advanced stoves							relative low emission levels					
	Ambitious pollution control	All region	-			cleaner coals and stoves			further reduce the ash and sulfur content in residential coal, and fully applied advanced stoves			relative low emission levels						Innovation of stoves and residential fuels			
Solvent use	Baseline	All region	-																		
	Current goals	BTHS & FWP	-			lower the VOCs content			further improve the water-soluble solvent use; install VOC control facility in coating and painting industry				relative low emission levels								
		YRD	-			lower the VOCs content			further improve the water-soluble solvent use; install VOC control facility in coating and painting industry					relative low emission levels							
		Other regions	-			lower the VOCs content			further improve the water-soluble solvent use; install VOC control facility in coating and painting industry							relative low emission levels					
	Ambitious pollution control	All region	-			lower the VOCs content			further improve the water-soluble solvent use; install VOC control facility in coating and painting industry				relative low emission levels					Innovation of solvent and VOC control facility			
Agriculture	Baseline	All region	-																		
	Current goals	BTHS & FWP	-			promote the use of organic fertilizer and the resource utilization of the poultry excrement and straw			enhance the intensive cutivation and grazierty; promote the slow-release fertilizer				relative low emission levels								
		YRD	-						enhance the intensive cutivation and grazierty; promote the slow-release fertilizer					relative low emission levels							
		Other regions	-						enhance the intensive cutivation and grazierty; promote the slow-release fertilizer							relative low emission levels					
	Ambitious pollution control	All region	-						enhance the intensive cultivation and graziery; promote the slow-release fertilizer				relative low emission levels					Innovation of cultivation and graziery			

Table S2. Descriptions of scenarios used in this study.

Scenario name	Socioeconomic assumptions	Climate targets	Air pollution control	Relationship with Tong's work	Scenario definition
Baseline	SSP4	RCP6.0	Baseline	SSP4-60-BAU	*Baseline* represents the baseline scenario and adapts to the SSP4 global developing modes. Inequality remains high and economies are relatively isolated, which leads the development in China to proceed slowly. China therefore would develop with a pessimistic trend, and the economic growth would slow down, which lead to limited actions on climate and environmental issues. Thus, the climate constraints would be negligible under RCP6.0 and the environmental control would remain as the 2015 level.
Current-goals	SSP2	RCP4.5	Current goals	SSP2-45-ECP	*Current-goals* is basically matched China's NDC carbon mitigation target and the national ambient air quality standards (i.e. 35 μg/m^3) by 2030, elucidating China's future emission, air quality evolutions towards released and upcoming policies, both on climate and pollution control. NDC-adapted energy transition would be performed, that China would achieve the carbon peak and the non-fossil fuel fractions would rise to ~18% in 2030. For air pollution control, key industries (i.e. iron, steel, cement, metal) would meet the special emission standards around 2020 according to China's clean air actions and further attain the ultra-low emission standards by 2030 based on the upcoming plans and the goal of 'a beautiful China'. However, during the post-2030 periods, no extra environment policies or technology innovations would be applied, and the energy transition basically follows the RCP4.5 trajectories. For socio-economic development, the central pathway of SSP2 is adopted, representing China's moderation future along with the historical patterns.
Ambitious-pollution-NDC-goals	SSP2	RCP4.5	Ambitious pollution control	Newly added	*Ambitious-pollution-NDC-goals* shares the same energy evolutions and economic pathway with the *Current-goals* scenario during 2015–2060. On the aspect of air pollution control, key industries would be upgraded to achieve the ultra-low emission standards comprehensively by 2030 along with China's medium-term environmental targets and modernization goals. And on post-2030 periods, the strengthened pollution control measures would be persisted, with even more optimistic long-term environmental policies, investments and the fully-applied best available technologies across all sectors by 2050. And these rigorous policies and best available technologies would be implemented consistently during 2050-2060, to achieve clean air as in developed countries and maximally protect human health.

Ambitious-pollution-Neutral-goals	SSP1	Net-zero CO_2 emission in 2060	Ambitious pollution control	Newly added	*Ambitious-pollution-Neutral-goals* is designed to pursue China's carbon neutrality goals and the WHO Air Quality Guideline (i.e. 10μg/m^3) from China's long-term air quality improvement perspectives by 2060. Rapid and ambitious low-carbon energy transitions would be deployed to ensure net-zero CO_2 emissions in 2060, with an average natural sink of ~0.7 Gt CO_2 in 2060 under the low radiation forcing scenarios (i.e. RCP2.6, RCP1.9) projected by published study[30] and our simulations (Table S3). Primary coal and oil fractions would be decreased below 9% and 10% respectively in 2060; while the low-carbon energy (i.e. solar, wind, biomass, nuclear energy) fractions would rise to ~72%. For the air pollution control, *Ambitious-pollution-Neutral-goals* shares the same end-of-pipe control policy and technology evolutions as *Ambitious-pollution-NDC-goals*, that the best available technologies would be entirely infiltrated among all sectors by 2050, and implemented consistently during 2050-2060. Meanwhile, following the heterogeneous and sustainable SSP1 pathways, China would develop sustainably at a reasonably high pace, with the rapid development of technology, economy, productivity, education. More finances would be invested in decarburization and decontamination toward a friendly environment and public health.
Ambitious-pollution-2℃-goals	SSP1	RCP2.6	Ambitious pollution control	SSP1-26-BHE	*Ambitious-pollution-2℃-goals* is designed to pursue the 2℃ temperature limits in Paris Agreement and the WHO Air Quality Guideline (i.e. 10μg/m^3) from China's long-term air quality improvement perspectives by 2060. The energy transition is compatible with 2℃ limits, however less than the SSP1-19 pathway. Primary coal and oil fractions would be decreased below 13% and 15% respectively in 2060; while the low-carbon energy (i.e. solar, wind, biomass, nuclear energy) fractions would rise to ~60%. For the air pollution control and socioeconomic developments, *Ambitious-pollution-2℃-goals* shares the same end-of-pipe control evolutions and SSP1 assumptions as *Ambitious-pollution-Neutral-goals*, that the best available technologies would be entirely infiltrated among all sectors by 2050, and implemented consistently during 2050-2060.

Ambitious-pollution-1.5℃-goals	SSP1	RCP1.9	Ambitious pollution control	Newly added	*Ambitious-pollution-1.5℃-goals* is designed to pursue the 1.5℃ global temperature rise limits and the WHO Air Quality Guideline (i.e. 10μg/m^3) by 2060. The most innovative and strictest co-control measures would be implemented. A low-carbon energy system would be established promptly with the pervasive electrification and widespread renewable applications; and traditional fossil fuels of coal and oil would be eliminated instantly. Primary coal and oil fractions would be decreased below 6% and 5% in 2060 respectively; while the low-carbon energy (i.e. solar, wind, biomass, nuclear energy) fractions would rise to ~82%. For the air pollution control and socioeconomic developments, *Ambitious-pollution-1.5℃-goals* shares the same end-of-pipe control evolutions and SSP1 assumptions as *Ambitious-pollution-Neutral-goals*, that the best available technologies would be entirely infiltrated among all sectors by 2050, and implemented consistently during 2050-2060. Given that the potential of available pollution control measures would be fully released under the maximum-control scenarios, further air quality progress would be mainly depended on clean energy transitions toward 1.5℃ climate target.

Table S3. China's anthropogenic CO_2 emissions under the NDC target in 2030 and the projected natural carbon sink in 2060 under low radiation forcing scenarios (applied as China's carbon neutrality goals in 2060).

Targets	Year	CO_2 emissions (2030) / natural sink (2060) (Gt CO_2)	Sources
China's NDC pledges	2030	11.4 – 14	Meinshausen, M. et al 2015[31]
		12.9 – 15.2	Pan et al., 2017[32]
		11.8	Gallagher et al., 2019[33]
		10.0 – 12.0	Chai et al., 2014[34]
		11.3 – 11.8	Elzen et al., 2016[35]
		10.0 – 12.5	Grubb et al., 2015[36]
		9.7 – 11.9	Zhou et al., 2013[37]
		10.6	He et al., 2014[38]
		10.2	Zhang et al., 2016[39]
		10.8 – 11.8	He et al., 2019
		13.3	CD-Links, Van deg Berg et al., 2019
		13.957	PBL; ISSIA, 2015
		11.0	Li et al., 2019[40]
China's carbon neutrality goals	2060	0.43-1.34	Wang et al., 2014[30]
		0.55	SSP1-26 simulation
		0.45	SSP1-19 simulation

Table S4. Region definitions in this study.

Region	Province / City inclusions
Beijing-Tianjin-Hebei and Surroundings (BTHS)[41]	Beijing city; Tianjin city; Shijiazhuang, Tangshan, Handan, Baoding, Langfang, Cangzhou, Hengshui, Xingtai city in Hebei Province; Taiyuan, Changzhi, Yangquan, Jincheng city in Shanxi Province; Zhengzhou, Anyang, Hebi, Kaifeng, Jiaozuo, Xinxiang, Puyang city in Henan Province; Jinan, Dezhou, Liaocheng, Binzhou, Zibo, Jining, Heze city in Shandong Province
Fenwei Plain (FWP)[41]	Xi'an, Xianyang, Tongchuan, Baoji, Weinan city in Shaanxi Province; Jinzhong, Linfen, Yuncheng, Lvliang city in Shanxi Province; Luoyang, Sanmenxia city in Henan Province
Yangtze River Delta (YRD)[41]	Shanghai city, Jiangsu, Zhejiang, Anhui Province
Sichuan Basin (SCB)[42]	Chengdu, Zigong, Luzhou, Deyang, Mianyang, Suining, Neijiang, Leshan, Meishan, Yibin, Ya'an, Ziyang, Nanchong, Guang'an, Dazhou city in Sichuan Province; Chongqin city
Pearl River Delta (PRD)[42]	Guangzhou, Shenzhen, Zhuhai, Foushan, Jiangmen, Zhaoqing, Huizhou, Dongguan, Zhongshan city in Guangdong Province

Table S5. Summary of emission and meteorology configurations used in this study.

Simulation type	Scenario	China's anthropogenic emission scenario and year	Global anthropogenic emission scenario and year	Natural source emission year	Chemical boundary	Meteorology year
Core simulation	Base-year (2015)	MEIC, 2015	SSP2-45, 2015	2015	GEOS-Chem outputs with global SSP2-45 emissions in 2015	2015
Core simulation	Baseline (2030)	Baseline, 2030	SSP4-60, 2030	2015	GEOS-Chem outputs with global SSP4-60 emissions in 2030	2015
Core simulation	Baseline (2060)	Baseline, 2060	SSP4-60, 2060	2015	GEOS-Chem outputs with global SSP4-60 emissions in 2060	2015
Core simulation	Current-goals (2030)	Current-goals, 2030	SSP2-45, 2030	2015	GEOS-Chem outputs with global SSP2-45 emissions in 2030	2015
Core simulation	Current-goals (2060)	Current-goals, 2060	SSP2-45, 2060	2015	GEOS-Chem outputs with global SSP2-45 emissions in 2060	2015
Core simulation	Ambitious-pollution-NDC-goals (2030)	Ambitious-pollution-NDC-goals, 2030	SSP2-45, 2030	2015	GEOS-Chem outputs with global SSP2-45 emissions in 2030	2015
Core simulation	Ambitious-pollution-NDC-goals (2060)	Ambitious-pollution-NDC-goals, 2060	SSP2-45, 2060	2015	GEOS-Chem outputs with global SSP2-45 emissions in 2060	2015
Core simulation	Ambitious-pollution-Neutral-goals (2030)	Ambitious-pollution-Neutral-goals, 2030	SSP1-26, 2030	2015	GEOS-Chem outputs with global SSP1-26 emissions in 2030	2015

Core simulation	Ambitious-pollution-Neutral-goals (2060)	Ambitious-pollution-Neutral-goals, 2060	SSP1-26, 2060	2015	GEOS-Chem outputs with global SSP1-26 emissions in 2060	2015
Core simulation	Ambitious-pollution-2℃-goals (2030)	Ambitious-pollution-2℃-goals, 2030	SSP1-26, 2030	2015	GEOS-Chem outputs with global SSP1-26 emissions in 2030	2015
Core simulation	Ambitious-pollution-2℃-goals (2060)	Ambitious-pollution-2℃-goals, 2060	SSP1-26, 2060	2015	GEOS-Chem outputs with global SSP1-26 emissions in 2060	2015
Core simulation	Ambitious-pollution-1.5℃-goals (2030)	Ambitious-pollution-1.5℃-goals, 2030	SSP1-19, 2030	2015	GEOS-Chem outputs with global SSP1-19 emissions in 2030	2015
Core simulation	Ambitious-pollution-1.5℃-goals (2060)	Ambitious-pollution-1.5℃-goals, 2060	SSP1-19, 2060	2015	GEOS-Chem outputs with global SSP1-19 emissions in 2060	2015
Sensitivity simulation	Meteorology sensitivity (2030)	Ambitious-pollution-2℃-goals, 2030	SSP1-26, 2030	2015	GEOS-Chem outputs with global SSP1-26 emissions in 2030	2000-2018 (a total of 19 years' simulations)
Sensitivity simulation	Meteorology sensitivity (2060)	Ambitious-pollution-2℃-goals, 2060	SSP1-26, 2060	2015	GEOS-Chem outputs with global SSP1-26 emissions in 2060	2000-2018 (a total of 19 years' simulations)
Sensitivity simulation	Transport sensitivity (2030)	Ambitious-pollution-2℃-goals, 2030	SSP3-70, 2030	2015	GEOS-Chem outputs with global SSP3-70 emissions in 2030	2015

Sensitivity simulation	Transport sensitivity (2060)	Ambitious-pollution-2℃-goals, 2060	SSP3-70, 2060	2015	GEOS-Chem outputs with global SSP3-70 emissions in 2060	2015
Sensitivity simulation	Transport sensitivity (2030)	Ambitious-pollution-2℃-goals, 2030	SSP1-19, 2030	2015	GEOS-Chem outputs with global SSP1-19 emissions in 2030	2015
Sensitivity simulation	Transport sensitivity (2060)	Ambitious-pollution-2℃-goals, 2060	SSP1-19, 2060	2015	GEOS-Chem outputs with global SSP1-19 emissions in 2060	2015
Sensitivity simulation	Natural source emission sensitivity	None	None	2015	Static default profile	2015

SI References

1 O'Neill BCT, Tebaldi C and van Vuuren DP *et al*. The Scenario Model Intercomparison Project (ScenarioMIP) for CMIP6. *Geosci. Model Dev.* 2016; **9:** 3461-3482.

2 Chou MD, Suarez MJ and Ho CH *et al.* Parameterizations for Cloud Overlapping and Shortwave Single-Scattering Properties for Use in General Circulation and Cloud Ensemble Models. *J. Climate* 1998; **11:** 202-214.

3 Mlawer EJ, Taubman SJ and Brown PD *et al.* Radiative transfer for inhomogeneous atmospheres: RRTM, a validated correlated-k model for the longwave. *J. Geophys. Res. Atmos.* 1997; **102:** 16663-16682.

4 Kain JS. The Kain–Fritsch Convective Parameterization: An Update. *J. Appl. Meteorol.* 2004; **43:** 170-181.

5 Pleim JE. A Combined Local and Nonlocal Closure Model for the Atmospheric Boundary Layer. Part II: Application and Evaluation in a Mesoscale Meteorological Model. *J. Appl. Meteorol.* 2007; **46:** 1396-1409.

6 Xiu AJ and Pleim JE. Development of a Land Surface Model. Part I: Application in a Mesoscale Meteorological Model. *J. Appl. Meteorol.* 2001; **40:** 192-209.

7 Mass C and Ovens D. WRF model physics: progress, prob-lems, and perhaps some solutions, The 11th WRF Users' Workshop, NCAR Center Green Campus, 21-25 June, 2010.

8 Zhang Q, Streets DG and Carmichael GR *et al*. Asian emissions in 2006 for the NASA INTEX-B mission, *Atmos. Chem. Phys.* 2009; **9:** 5131–5153.

9 Feng L, Smith SJ and Craun C *et al.* The generation of gridded emissions data for CMIP6. *Geosci. Model Dev.* 2020; **13:** 461-482.

10 Guenther AB, Jiang X and Heald CL *et al.* The Model of Emissions of Gases and Aerosols from Nature version 2.1 (MEGAN2.1): an extended and updated framework for modeling biogenic emissions. *Geosci. Model Dev.* 2012; **5:** 1471-1492.

11 van der W, Guido R and Randerson JT *et al.* Global fire emissions estimates during 1997–2016. *Earth Syst. Sci. Data* 2017; **9:** 697-720.

12 Xue T, Zheng YX and Tong D *et al*. Spatiotemporal continuous estimates of $PM_{2.5}$ concentrations in China, 2000-2016: A machine learning method with inputs from satellites, chemical transport model, and ground observations. *Environ. Int.* 2019; **123:** 345-357.

13 Xue T, Zheng YX and Geng GN *et al.* Fusing Observational, Satellite Remote Sensing and Air Quality Model Simulated Data to Estimate Spatiotemporal Variations of $PM_{2.5}$ Exposure in China. *Remote Sens*. 2017; **9:** 221.

14 Dong XY, Li J and Fu JS *et al.* Inorganic aerosols responses to emission changes in Yangtze River Delta, China. *Sci. Total Environ.* 2014; **481:** 522-532.

15 Megaritis AG, Fountoukis C and Charalampidis PE *et al.* Response of fine particulate matter concentrations to changes of emissions and temperature in Europe. *Atmos. Chem. Phys.* 2013; **13:** 3423–3443.

16 Brasseur GP and Jacob DJ. Modeling of Atmospheric Chemistry. Cambridge University Press, 2017.

17 Emery C, Liu Z and Russell AG *et al.* Recommendations on statistics and benchmarks to assess photochemical model performance, *J. Air Waste Manage. Assoc.* 2017; **67:** 582-598.

18 Li HY, Cheng J and Zhang Q *et al.* Rapid transition in winter aerosol composition in Beijing from 2014 to 2017: response to clean air actions. *Atmos. Chem. Phys.* 2019; **19:** 11485–11499.

19 Zhang Q, Zheng YX and Tong D *et al*. Drivers of improved $PM_{2.5}$ air quality in China from 2013 to 2017. *Proc. Natl Acad. Sci. USA* 2019; **116:** 24463-24469.

20 Zhong Q, Ma JM and Shen GF *et al.* Distinguishing Emission-Associated Ambient Air $PM_{2.5}$ Concentrations and Meteorological Factor-Induced Fluctuations. *Environ. Sci. Technol.* 2018; **52:** 10416-10425.

21 Han LH, Cheng SY and Zhuang GS *et al*. The changes and long-range transport of $PM_{2.5}$ in Beijing in the past decade. *Atmos. Environ.* 2015; **110:** 186-195.

22 Shen L, Jacob DJ and Mickley LJ *et al*. Insignificant effect of climate change on winter haze pollution in Beijing. *Atmos. Chem. Phys*. 2018; **18:** 17489–17496.

23 Hong CP, Zhang Q and Zhang Y *et al.* Impacts of climate change on future air quality and human health in China. *Proc. Natl Acad. Sci. USA* 2019; **116:** 17193–17200.

24 Cai W, Li K and Liao H *et al.* Weather conditions conducive to Beijing severe haze more frequent under climate change. *Nat. Clim. Chang.* 2017; **7:** 257-262.
25 Wang HJ, Chen HP and Liu JP. Arctic sea ice decline intensified haze pollution in eastern China, *Atmos. Ocean. Sci. Lett*. 2015; **8:** 1–9.
26 Tegen I, Werner M and Harrison SP *et al*. Relative importance of climate and land use in determining present and future global soil dust emission. *Geophys. Res. Lett*. 2004; **31:** L05105.
27 Tsunematsu N, Kuze H and Sato T *et al*. Potential impact of spatial patterns of future atmospheric warming on Asian dust emission. *Atmos. Environ.* 2011; **45:** 6682-6695.
28 Fu Y and Liao H. Impacts of land use and land cover changes on biogenic emissions of volatile organic compounds in China from the late 1980s to the mid-2000s: implications for tropospheric ozone and secondary organic aerosol. *Tellus B* 2014; **66:** 24987.
29 Liu S, Xing J and Zhang HL *et al.* Climate-driven trends of biogenic volatile organic compound emissions and their impacts on summertime ozone and secondary organic aerosol in China in the 2050s. *Atmos. Environ.* 2019; **218:** 117020.
30 Wang T, Lin X and Peng SS *et al*. Multimodel projections and uncertainties of net ecosystem production in China over the twenty-first century. *Chin. Sci. Bull.* 2014; **59 (34):** 4681–4691.
31 Meinshausen M, Jeffery L and Guetschow J *et al.* National post-2020 greenhouse gas targets and diversity-aware leadership. *Nat. Clim. Chang.* 2015; **5:**1098-1106.
32 Pan X, Elzen MD and Höhne N *et al.* Exploring fair and ambitious mitigation contributions under the Paris Agreement goals. *Environ. Sci. Policy* 2017; **74:** 49-56.
33 Gallagher KS, Zhang F and Orvis R *et al.* Assessing the Policy gaps for achieving China's climate targets in the Paris Agreement. *Nat. Commun.* 2019; **10:** 1256.
34 Chai Q and Xu H. Modeling an emissions peak in China around 2030: Synergies or trade-offs between economy, energy and climate security. *Adv. Clim. Chang. Res.* 2014; **5:** 169-180.
35 Elzen MD, Fekete H and Höhne N *et al.* Greenhouse gas emissions from current and enhanced policies of China until 2030: Can emissions peak before 2030? *Ener. Policy* 2016; **89:** 224-236.
36 Grubb M, Fu S and Spencer T *et al.* A review of Chinese CO_2 emission projections to 2030: the role of economic structure and policy. *Clim. Policy* 2015; 15: S7-S39.
37 Zhou N, Fridley D and Khanna NZ *et al.* China's energy and emissions outlook to 2050: Perspectives from bottom-up energy end-use model. *Ener. Policy* 2013; **53:** 51-62.
38 He JK. Analysis of CO_2 emissions peak: China’s objective and strategy. *China Popul. Resour. Environ.* 2014; **12:** 189-198.
39 Zhang XL, Karplus VJ and Qi T *et al.* Carbon emissions in China: How far can new efforts bend the curve? *Energy Econ.* 2016; **54:** 388-395.
40 Li N, Chen WY and Rafaj P *et al*. Air Quality Improvement Co-benefits of Low-Carbon Pathways toward Well Below the 2 degrees C Climate Target in China. *Environ. Sci. Technol.* 2019; **53:** 5576-5584.
41 State Council of the People’s Republic of China. *Notice of the state council on issuing the three-year action plan for winning the Blue Sky defense battle.* Beijing: China Environmental Science Press, 2018.
42 China National Environmental Monitoring Centre. *National Urban Air Quality Report*.
